# Oxidative Stress and Microvessel Barrier Dysfunction

**DOI:** 10.3389/fphys.2020.00472

**Published:** 2020-05-27

**Authors:** Pingnian He, M. A. Hassan Talukder, Feng Gao

**Affiliations:** Department of Cellular and Molecular Physiology, College of Medicine, The Pennsylvania State University, Hershey, PA, United States

**Keywords:** reactive oxygen species, reactive nitrogen species, microvessel permeability, pericyte, Nrf-2

## Abstract

Clinical and experimental evidence indicate that increased vascular permeability contributes to many disease-associated vascular complications. Oxidative stress with increased production of reactive oxygen species (ROS) has been implicated in a wide variety of pathological conditions, including inflammation and many cardiovascular diseases. It is thus important to identify the role of ROS and their mechanistic significance in microvessel barrier dysfunction under pathological conditions. The role of specific ROS and their cross talk in pathological processes is complex. The mechanisms of ROS-induced increases in vascular permeability remain poorly understood. The sources of ROS in diseases have been extensively reviewed at enzyme levels. This review will instead focus on the underlying mechanisms of ROS release by leukocytes, the differentiate effects and signaling mechanisms of individual ROS on endothelial cells, pericytes and microvessel barrier function, as well as the interplay of reactive oxygen species, nitric oxide, and nitrogen species in ROS-mediated vascular barrier dysfunction. As a counter balance of excessive ROS, nuclear factor erythroid 2 related factor 2 (Nrf2), a redox-sensitive cell-protective transcription factor, will be highlighted as a potential therapeutic target for antioxidant defenses. The advantages and limitations of different experimental approaches used for the study of ROS-induced endothelial barrier function are also discussed. This article will outline the advances emerged mainly from *in vivo* and *ex vivo* studies and attempt to consolidate some of the opposing views in the field, and hence provide a better understanding of ROS-mediated microvessel barrier dysfunction and benefit the development of therapeutic strategies.

## Introduction

Oxidative stress with increased production of reactive oxygen species (ROS) has been implicated in a wide variety of pathological conditions, such as inflammation, atherosclerosis, ischemia-reperfusion injury, hypercholesterolemia, hypertension, diabetes, and heart failure (Mcquaid and Keenan, 1997; Lum and Roebuck, 2001; Harrison et al., 2003; Stocker and Keaney, 2004; Forstermann, 2008; Li and Forstermann, 2013; Kalogeris et al., 2016; Forstermann et al., 2017; Zhou et al., 2019). Growing evidence indicates that overproduction of ROS can lead to endothelial barrier dysfunction with subsequent increased vascular permeability, tissue damage, and organ dysfunction (Del Maestro et al., 1981; Cai and Harrison, 2000; Henricks and Nijkamp, 2001; Zhu and He, 2006; Hecquet et al., 2008; Zhou et al., 2009, 2013, 2019). However, the precise role played by each ROS and their products in normal and pathological processes is very complex, and the mechanisms of ROS-induced increased vascular permeability under inflammation and disease conditions remain largely unknown. This review will discuss the sources of ROS and antioxidants in the vasculature with focus on the effects and mechanisms of different ROS and ROS-derived reactive nitrogen species (RNS) on endothelial barrier function. The advantages and limitations of different experimental approaches used for the study of ROS-induced endothelial barrier function will also be discussed in this review.

## Sources of ROS, Redox-Crosstalk, and Antioxidants in Vascular System

Reactive oxygen species are a collective term that refers to both oxygen-derived unstable free radicals, such as superoxide anion, hydroxyl radical, oxygen radical, NO, and non-free radicals, such as hydrogen peroxide (H_2_O_2_), hypochlorus acid, peroxynitrite (ONOO^–^), and ozone (Cai and Harrison, 2000; Madamanchi et al., 2005; Forstermann, 2008; Forstermann et al., 2017). ROS are ubiquitous and formed continuously in small amounts during the normal metabolism of cells and are normally inactivated by endogenous antioxidant defense mechanisms (Lum, 2001; Zweier and Talukder, 2006). The sources of different ROS and antioxidant enzymes in vascular diseases have been extensively reviewed by several authors (Cai and Harrison, 2000; Madamanchi et al., 2005; Mueller et al., 2005; Forstermann, 2008; Montezano and Touyz, 2012; Kalogeris et al., 2016; Forstermann et al., 2017). The major sources of ROS in ECs are from NADPH oxidase (Nox), xanthine oxidase (XO), mitochondria, and dysfunctional eNOS. Other potential vascular sources of ROS include cytochrome p450 mono-oxygenase, cyclo-oxygenase and lipoxygenase, NOS, and peroxidases (Forstermann, 2008). In addition to the ROS derived from activated ECs, the vascular endothelium is constantly exposed to various blood-borne agents and is the primary target for oxidants released from activated blood cells during inflammation. The activated neutrophils could release large amount of ROS to the circulation via membrane-bound NADPH oxidase during neutrophil respiratory burst, which provide the killing power for invading pathogens, but also cause host tissue injury and endothelial barrier dysfunction (Carden et al., 1990; Babior, 1999; Dahlgren and Karlsson, 1999; Kubes et al., 1990a; Madamanchi et al., 2005; He et al., 2006; Zhu and He, 2006; Forstermann, 2008). Importantly, there appears to be significant redox crosstalk between these pro-oxidant systems where ROS produced by one enzyme system can enhance the activity of other ROS-producing systems, leading to feed-forward processes with augmented ROS production and oxidative stress (Cai, 2005b; Forstermann, 2008; Daiber, 2010; Zinkevich and Gutterman, 2011; Daiber et al., 2017; Zhou et al., 2019). For instance, NADPH oxidase-derived ROS have been implicated in the activation of XO (Mcnally et al., 2003; Landmesser et al., 2007), mitochondrial ROS production (Wenzel et al., 2008; Kroller-Schon et al., 2014), uncoupling of eNOS (Landmesser et al., 2003), and NO/O_2_^–^ derived ONOO^–^ formation (Radi et al., 1991; Xia et al., 1996; Riobo et al., 2001; Pacher et al., 2007; Szabo et al., 2007; Zhou et al., 2019).

Reactive oxygen species in the cells and tissues are kept in balance by the anti-oxidant enzymes that regulate and often reduce the level of ROS in biological system. Vascular cells are equipped with several of these protective enzymes such as superoxide dismutase (SOD), catalase, glutathione peroxidase, thioredoxin, heme oxygenase, and paraoxonase (Forstermann, 2008). Several non-enzymatic antioxidants are also found in the biological systems such as endogenous glutathione, uric acid and bilirubin, and ingested antioxidant vitamins (Forstermann, 2008). While ROS at moderate and low concentrations have important signaling roles and beneficial effects under physiological conditions (Suzuki et al., 1997; Finkel, 1998; Kamata and Hirata, 1999; Rhee, 1999; Thannickal and Fanburg, 2000; Droge, 2002; Valko et al., 2007), at higher concentrations, especially with reduced antioxidant counter balance, ROS generate oxidative stress under pathophysiologic conditions causing potential damage to the biomolecules, disruption of cellular function and promoting disease progression (Yla-Herttuala, 1999; Stadtman and Levine, 2000; Lum, 2001; Madamanchi et al., 2005; Pacher et al., 2007; Szabo et al., 2007; Forstermann, 2008; Li et al., 2014). Although ample experimental evidence support the role of certain antioxidant enzymes or non-enzymatic antioxidants in ameliorating oxidative stress-mediated vascular damages, clinical studies so far have not been able to demonstrate conclusive results (Madamanchi et al., 2005; Landmesser et al., 2007; Forstermann, 2008; Boueiz and Hassoun, 2009). These led more studies to focus on the transcriptional regulations of antioxidant genes. Among varieties of redox-sensitive transcription factors, nuclear factor (erythroid 2–related) factor 2 (Nrf2) has recently been drawn attention and referred to as master antioxidant that regulates a wide variety of genes encoding detoxification enzymes and antioxidant proteins (Ma, 2013; Chen et al., 2015; Amin et al., 2019). Recent development of the role of Nrf2 in the regulation of redox homeostasis and its therapeutic prospects are further discussed in the later section.

## Leukocyte-Dependent ROS-Mediated Microvascular Barrier Dysfunction

Acute inflammation is characterized by increased microvascular permeability to plasma proteins and leukocyte recruitment into inflammatory sites, which occurs mainly at post-capillary venules. The recruited granulocytes upon activation release various substances including ROS during respiratory bust. Due to its large quantity over other sources, ROS have been demonstrated to play a major role in the acute activation of endothelial cells, resulting in vascular leakages (Del Maestro et al., 1981; Zhu et al., 2005; Zhu and He, 2006; Granger and Kvietys, 2015). The leukocyte-dependent ROS-mediated microvascular barrier dysfunction was supported by experimental evidence that the blocking antibodies, induction of neutropenia, or the application of superoxide scavenger SOD reduced vascular damages in reperfusion of ischemic tissues and other inflammatory conditions (Korthuis et al., 1988; Carden et al., 1990; Kadambi and Skalak, 2000; Granger and Kvietys, 2015).

### Role of Leukocyte Adhesion/Emigration in Microvessel Permeability

Leukocyte adhesion, as a necessary step leading to granulocytes migration from the vascular lumen to the interstitial space, has been indicated as a prerequisite for leukocyte to induce endothelial injury in studies conducted in autologous blood perfused organ or tissues (Carden et al., 1990; Kubes et al., 1990a). Supporting this notion, some *in vitro* studies reported that the adhesion process could directly trigger intracellular signals in ECs or protease release from neutrophils (Moll et al., 1998) resulting in increased EC permeability (Del Maschio et al., 1996). However, when vascular permeability and leucocyte/endothelium interactions were evaluated simultaneously in vasculature, the sites of the leucocyte adhesion and emigration were not always associated with vascular leakages (Hurley, 1964; Mcdonald, 1994; Baluk et al., 1995; Zeng et al., 2002). Multiple studies revealed temporal and spatial dissociations of leucocyte adhesion and/or emigration from vascular leakages, indicating that mechanisms other than the adhesion process could be critical to the vascular barrier dysfunction during inflammation (Harris et al., 1993; Kadambi and Skalak, 2000; Zeng et al., 2002; He, 2010).

### Role of Neutrophil Released ROS in Microvessel Permeability

**FIGURE 1:**
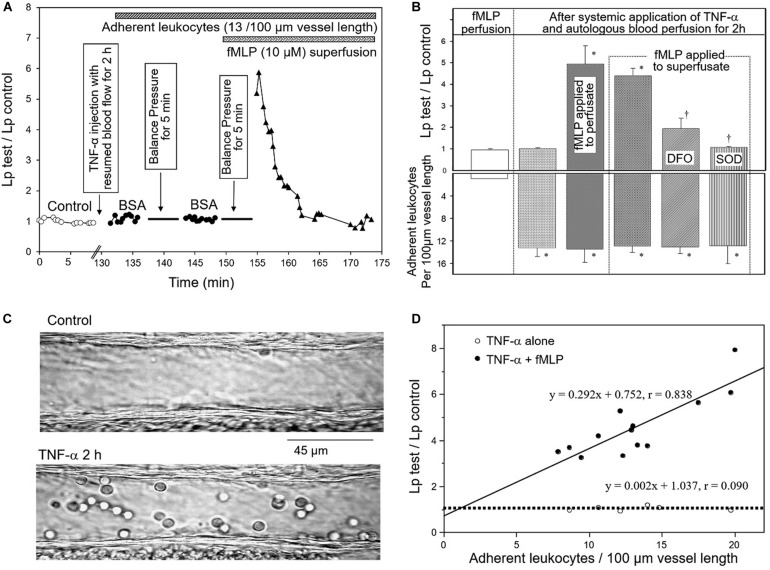
TNF-α-induced adherent leukocytes do not increase microvessel permeability until exposure to fMLP. **(A)** Lp measurements in an individually perfused rat mesenteric venule. The time course of the measured Lp shows that neither TNF-α-induced leukocyte adhesion alone nor stationary flow in the presence of adherent leukocytes (to allow accumulation of locally released agents) changed Lp. Transient increase in Lp only occurred after adherent leukocytes were exposed to fMLP (10 μmol/L). **(B)** Summary results showing relationship between number of adherent leukocytes (bottom graph) and changes in microvessel Lp (top graph). Significant increases in Lp only occurred after fMLP was applied to perfusate or superfusate, and was attenuated or prevented by pretreatment of the vessel with an iron chelator DFO or SOD, indicating the role of released ROS in increased Lp. **(C)** Images of individually perfused venules under control conditions and after TNF-α-induced leukocyte adhesion (2 h after systemically applied TNF-α). **(D)** The positive correlation between the increased Lp and the number of adherent leukocytes with fMLP stimulation (•) indicates that the quantity of fMLP-induced release of ROS correlated with the number of adherent leukocytes determines the magnitude of Lp increases. Modified and used with permission (Zhu and He, 2006).

Whether leucocyte/endothelium interactions would be the cause of increased microvessel permeability has been controversial for decades (Carden et al., 1990; Kubes et al., 1990b; Baluk et al., 1997; Moll et al., 1998; Zeng et al., 2002; He, 2010). On one hand, studies reported leucocyte adhesion and emigration to be the critical event leading to tissue and organ dysfunction during inflammation, and the number of adherent leucocytes was considered as an index for the severity of tissue injury and vascular dysfunction (Tailor and Granger, 2003; Ishikawa et al., 2004). On the other hand, experimental evidence showed that the albumin leakage sites were often distinct from those of leucocyte adhesion and emigration, indicating independent mechanisms to be involved in the induction of leucocyte adhesion and increased microvessel permeability (Hurley, 1964; Suematsu et al., 1993; Baluk et al., 1998; Zeng et al., 2002). Studies conducted in intact microvessels by dissecting the processes clarified some of the debating issues derived from different experimental approaches. The experiments shown in Figure 1 demonstrated that the systemic application of TNF-α causes a significant number of leucocyte adhesion at vascular walls, but no increased microvessel permeability was observed (Zeng et al., 2002). However, increased permeability occurred after fMLP was applied to TNF-α-induced adherent leukocytes and the application of SOD blocked such increased permeability, demonstrating ROS-mediated permeability increases. Importantly, in the presence of fMLP, the magnitude of the permeability increases was directly correlated with the number of adherent of leukocytes, indicating that larger quantity of released ROS produced by higher number of adherent leukocytes results in higher magnitude of permeability increases (Zhu and He, 2006). Figure 1D basically shows ROS dose-dependent permeability increases in intact microvessels. These observations suggest that in the presence of activating stimuli for leukocyte respiratory burst, the number of adherent leukocytes could predict the severity of vascular dysfunction as others concluded (Tailor and Granger, 2003; Ishikawa et al., 2004).

Although neutrophil/endothelium interactions is an essential part of acute inflammatory responses, a significant portion of responsive leucocytes remains in the peripheral circulation during inflammation. It is well known that isolated neutrophils could release large quantities of ROS through respiratory burst in response to a variety of stimuli, which are detectable by chemiluminescence measurement (Condliffe et al., 1998b; Babior, 1999). In addition to ROS-mediated permeability increased through adherent leukocyte release of ROS, studies showed that fMLP- or C5a-activated non-adherent neutrophils (suspension in the perfusate) could also increase permeability in isolated coronary venules (Tinsley et al., 2002) and intact mesenteric venules (Zhu et al., 2005). These experimental evidence strongly support that ROS released from activated leukocytes, in either circulating or adherent form, constitute the major source of ROS in the plasma during inflammation and disease conditions, which could directly activate ECs lining the vascular walls (details discussed below), contributing to leukocyte-dependent increases in vascular permeability. These results also suggested that the physical interaction between leukocytes and endothelium through adhesion/emigration was not the direct cause of vascular leakages, and the adhesion process was also not the direct trigger for neutrophils to release ROS.

### Neutrophil Priming and ROS Generation

**FIGURE 2:**
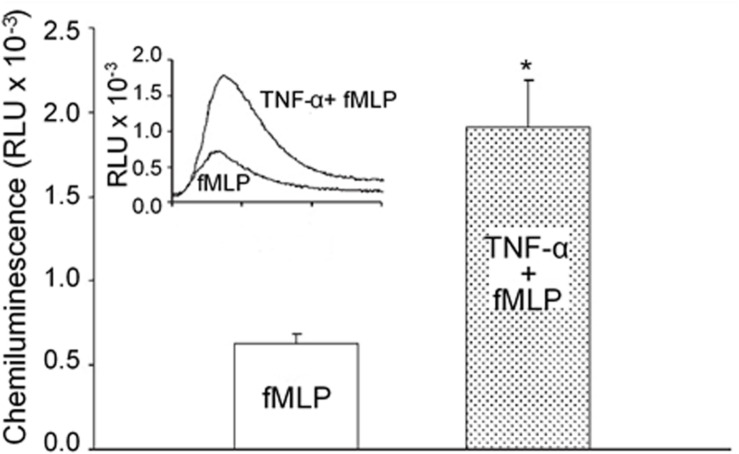
Priming effect of TNF-α on fMLP-stimulated neutrophil respiratory burst measured by chemiluminescence (CL). Preexposure of neutrophils to TNF-α through systemic application *in vivo* did not change basal CL but potentiated fMLP-stimulated CL by 3.1-fold. Bar graph shows magnitude difference in fMLP-stimulated CL between unprimed and TNF-α-primed neutrophils through *in vivo* application. Inset: representative individual time course from each group of experiments. Used with permission (Zhu and He, 2006). *Significant difference from fMLP stimulation alone.

Many cytokines and pro-inflammatory mediators have been reported as neutrophil primers that do not present the effector function on their own, but enable neutrophils to have enhanced responses to additional activating stimuli (Condliffe et al., 1998a; van Leeuwen et al., 2005; Zhu and He, 2006; El-Benna et al., 2016; Miralda et al., 2017). As shown in Figure 2, pre-exposure of neutrophils to a common priming cytokine, TNF-α, through systemic application *in vivo* did not induce ROS release as measured by chemiluminescence activity, but markedly augmented superoxide production in response to other mediators, such as fMLP (Zhu and He, 2006). The application of anti-TNF MoAb blocked TNF-α-mediated neutrophil priming (van Leeuwen et al., 2005). Under *in vivo* pathological conditions, systemically elevated cytokine levels or other priming agents such as chemoattractants, or microbial products could transform neutrophils into a primed state resulting in potentiated ROS release in responses to additional stimuli (Condliffe et al., 1998b; El-Benna et al., 2016; Miralda et al., 2017). Neutrophil priming with increased quantity of released ROS plays an important role in host defense against microbial pathogens, but also increases the risk for extensive tissue damage and endothelial barrier dysfunction. Severe clinical conditions such as adult respiratory distress syndrome (Parsons et al., 1989), organ failure, and mortality (Pinsky et al., 1993) are often associated with high plasma levels of inflammatory cytokines and/or endotoxin, and those stimuli-primed neutrophils with enhanced respiratory burst may explain the exacerbated tissue damages commonly observed in those conditions. Recent studies also suggest that primed neutrophils, in addition to enhanced oxidative burst, also involve activations of other neutrophil activities such as adhesion, surface receptor expression, etc (Miralda et al., 2017). Neutrophil priming and de-priming as well as the levels of the priming are complex processes which vary depending on the concentration of priming agents, exposure time, and the type of priming agents (Potera et al., 2016; Mcleish et al., 2017). The major priming agents, underlying mechanisms, and signaling pathways involved in priming neutrophil respiratory burst have been reviewed elsewhere in detail (Condliffe et al., 1998b; El-Benna et al., 2016; Miralda et al., 2017). In contrast to the priming agents that enhance the neutrophil responsiveness to stimuli, there are also cytokines that suppress neutrophil bacterial killing power with reduced ROS release. Multiple studies demonstrated that resistin, a cytokine commonly increased in chronic kidney diseases or sepsis, selectively inhibits neutrophil oxidative burst in response to stimuli and impair its bacterial killing capacity, resulting in immunosuppression (Cohen et al., 2008; Bonavia et al., 2017; Miller et al., 2019). A better understanding of the neutrophil priming *in vivo* under disease conditions may benefit the development of therapeutic strategies to minimize vascular damage, while enhancing its oxidative killing power at the bacterial battle field.

## Leukocyte-Derived Myeloperoxidase and Hypohalous Acids in Endothelial Dysfunction and Cardiovascular Diseases

Neutrophils in response to stimuli not only release ROS but also release myeloperoxidase (MPO), an enzyme closely linked to both inflammation and oxidative stress (Anatoliotakis et al., 2013; Ndrepepa, 2019). MPO is a member of the heme peroxidase-cyclooxygenase super family (Klebanoff, 2005; Davies et al., 2008; van der Veen et al., 2009; Davies, 2011). It is released by activated neutrophils, monocytes, and some tissue macrophages into extracellular spaces, where it catalyzes the conversion of hydrogen peroxide to hypohalous acids in the presence of halide and pseudo-halide ions (van Dalen et al., 1997; Hampton et al., 1998). This enzyme is abundantly expressed in granulocytes and accounts for approximately 5% of a neutrophil’s dry weight (Schultz and Kaminker, 1962; Davies, 2011). The major reactive species produced by MPO under physiological conditions are hypochlorous acid and hypothiocyanous acid. While hypohalous acids have potent antibacterial, antiviral, and antifungal properties and play key roles in the human immune system (Hampton et al., 1998; Barrett and Hawkins, 2012), an excessive recruitment and activation of leukocytes in the affected organ(s) under inflammatory conditions can amplify the inflammatory response and contribute to organ injury and dysfunction (Zhang C. et al., 2001; Sugiyama et al., 2004; Klebanoff, 2005; Lau and Baldus, 2006; Davies et al., 2008; Barrett and Hawkins, 2012). MPO-derived oxidants have been shown to damage vascular ECs, basement membrane and matrix components, and lead to leakage of plasma proteins, microvascular hemorrhage, and atherogenesis (Lush and Kvietys, 2000; Yang et al., 2006; Nicholls and Hazen, 2009; Wong et al., 2009; Tiyerili et al., 2016; Yu et al., 2019). MPO and its oxidant end products have been found in both human and experimental atherosclerotic lesions (Daugherty et al., 1994; van Leeuwen et al., 2008; La Rocca et al., 2009), and MPO-induced nitrotyrosine (NO_2_Tyr) formation in the vessel wall affects the matrix protein structure and function (Lau and Baldus, 2006). Some studies reported that MPO directly modulates endothelial NO production, NO bioavailability, and endothelium-dependent vasodilatation during acute inflammation (Zhang C. et al., 2001; Eiserich et al., 2002; Lau and Baldus, 2006). MPO-derived oxidants have also been found to inhibit sarco/endoplasmic reticulum Ca^2+^-ATPase activity, perturb Ca^2+^ homeostasis, resulting in the accumulation of cytosolic Ca^2+^ (Cook et al., 2012), caspase-3 activation and apoptosis of ECs in culture (Sugiyama et al., 2004). The direct effect of hypochlorous acid (HOCL) on endothelial permeability has been evaluated in cultured bovine pulmonary artery EC monolayers (Tatsumi and Fliss, 1994; Ochoa et al., 1997). Results showed that HOCl, either given directly or produced by conversion of H_2_O_2_ with MPO and Cl^–^, caused immediate changes in EC shape (cell retraction), electrical resistance, and increases in protein permeability (^125^I-labeled albumin clearance). The HOCl-mediated responses were shown to be faster and greater than those induced by H_2_O_2_ (Ochoa et al., 1997). The HOCl (10 to 100 μM)-induced increases in EC monolayer permeability occurred within 1–3 min, whereas H_2_O_2_-induced permeability increases in the same type of cultured ECs required higher concentration (50 to 400 μM) and longer exposure time (over 30 min), indicating that HOCI is a more potent oxidant than H_2_O_2_ to disrupt endothelial barrier function (Ochoa et al., 1997). Additionally, the MPO and MPO-catalyzed HOCI amplified high glucose-induced endothelial dysfunction in cell culture and rat aorta (Tian et al., 2017). Excessive production of hypohalous acids has been shown to be directly linked to many cardiovascular diseases (Zhang R. et al., 2001; Anatoliotakis et al., 2013; Ndrepepa, 2019) and circulating levels of MPO is clinically used as a biomarker to predict the health outcomes in those patients (Baldus et al., 2003; Brennan et al., 2003; Meuwese et al., 2007; Mocatta et al., 2007; Schindhelm et al., 2009). However, despite the growing evidence and interest in MPO-derived oxidant-induced cellular and molecular damage, the role of different MPO-derived species and mechanisms responsible for increased cardiovascular disease risk and patient outcomes are poorly defined and need further elucidation in detail (Davies, 2011; Barrett and Hawkins, 2012; Ndrepepa, 2019).

## ROS-Mediated Microvessel Barrier Dysfunction

Reactive oxygen species are derived from molecular oxygen by sequential monovalent reductions and the essential step in this process is the univalent enzymatic reduction of oxygen to superoxide radical (Del Maestro et al., 1981; Dreher and Junod, 1995; Babior, 1999; Forstermann, 2008). Superoxide in the extracellular space can spontaneously dismutate into H_2_O_2_ and O_2_, and the simultaneous presence of superoxide, H_2_O_2_, and chelated metal catalysts in the extracellular space can result in the generation of more active hydroxyl radical. Superoxide is unstable with a lifetime of few milliseconds at neutral pH, and hydroxyl is extremely reactive and short-lived free radical produced in biological systems (Zweier and Talukder, 2006; Montezano and Touyz, 2012).

Among ROS, H_2_O_2_ is particularly important in microvascular injury because it is relatively stable and has longer half-life than superoxide, and it is easily diffusible within and between cells (Montezano and Touyz, 2012). In light of the sources of *in vivo* oxidative stress, *in vitro* oxidative stress can be produced by direct application of oxidants such as H_2_O_2_ or continuous generation of superoxide or H_2_O_2_ in enzymatic systems (Dreher and Junod, 1995). One of the most widely used models of oxidative stress is the hypoxanthine (HX)/XO enzyme system (HX/XO) where XO acting aerobically on HX produces superoxide and H_2_O_2_ (Del Maestro et al., 1981; Dreher and Junod, 1995). While there is no clear evidence for the effects of blockade of production or bioactivity of endogenous oxidants on basal microvascular permeability (Kvietys and Granger, 2012), the reduction of endogenous NO has been shown to cause an immediate increase in ICAM-1-mediated leukocyte adhesion (Xu et al., 2013; Gao et al., 2017). Both *in vitro* and *in vivo* studies demonstrated that exogenous administration of H_2_O_2_ or HX/XO system can directly cause oxidative stress and increase the permeability of endothelial monolayers (Shasby et al., 1985; Siflinger-Birnboim et al., 1992; Berman and Martin, 1993; Doan et al., 1994; Okayama et al., 1999), individually perfused intact microvessels (Zhu and He, 2006; Zhou et al., 2009, 2013), whole vascular beds (Del Maestro et al., 1981; Parks et al., 1984), and the isolated organs (Johnson et al., 1989; Barnard and Matalon, 1992; Seeger et al., 1995). The protective effect of antioxidants (such as superoxide dismutase, catalase, XO inhibitor) against the permeability increases in different experimental models (Del Maestro et al., 1981; Parks et al., 1984; Shasby et al., 1985; Kennedy et al., 1989; Okayama et al., 1999; Zhou et al., 2009) and clinical study (Bernard et al., 1997) further implicated the role of oxidative stress in endothelial barrier dysfunction. Importantly, some studies in cultured ECs and intact microvessels have demonstrated that superoxide and H_2_O_2_ exert different effects on the endothelial permeability (Berman and Martin, 1993; Mcquaid and Keenan, 1997; Okayama et al., 1999; Zhou et al., 2009) with different cellular mechanisms (Mcquaid et al., 1996; Okayama et al., 1999; Zhou et al., 2013). Details are discussed below.

### Differential Effects of Superoxide and H_2_O_2_ on EC [Ca^2+^]_i_ and Microvessel Permeability

A number of *in vitro* studies in cultured monolayers of ECs from different origin have demonstrated that H_2_O_2_ can disrupt endothelial barrier function in a concentration-dependent manner with variable magnitudes (Shasby et al., 1985; Siflinger-Birnboim et al., 1992, 1996; Berman and Martin, 1993; Doan et al., 1994), and oxidant-mediated endothelial barrier dysfunction could be partially reversed and can occur in the absence of cytotoxicity (Shasby et al., 1985; Mcquaid et al., 1996; Gupta et al., 1998). H_2_O_2_-induced permeability increases in cultured ECs (measured by different methods) were reported to be rapid (Mcquaid et al., 1996; Hecquet et al., 2008) or delayed (Berman and Martin, 1993; Okayama et al., 1999). Similarly, both early and delayed permeability increases were also reported for superoxide generated by HX-XO in cultured EC monolayers (Berman and Martin, 1993; Okayama et al., 1999). The results of these *in vitro* studies were quite different and could be related to different species or culture conditions of the ECs or to the method differences in permeability measurements.

In intact microvessel studies the inflammatory mediator-induced increases in microvessel permeability are mediated by transiently increased EC [Ca^2+^]_i_ (Az-Ma et al., 1996; Zhu and He, 2005; Zhou and He, 2011). ROS has also been shown to induce increases in EC [Ca^2+^]_i_ by many *in vitro* and *in vivo* studies with exogenously applied ROS as stimuli (Doan et al., 1994; Volk et al., 1997; Hecquet et al., 2008) which correlates with increased endothelial permeability (Shasby et al., 1985; Siflinger-Birnboim et al., 1996; Zhu and He, 2006; Zhou et al., 2009, 2013). Superoxide, H_2_O_2_, and hydroxyl radical have been shown to exert differential effects on [Ca^2+^]_i_ in human ECs (Dreher and Junod, 1995). Importantly, it is reported that the toxicity of high levels of ROS could lead to a massive, steady influx of extracellular Ca^2+^, whereas low concentrations of ROS induce only transient Ca^2+^ changes, thus acting as signaling agonists (Volk et al., 1997). Certain *in vitro* studies reported rapid and transient increases in EC [Ca^2+^]_i_ when ECs were exposed to H_2_O_2_ (Siflinger-Birnboim et al., 1996; Volk et al., 1997), similar to those found with applied superoxide derived by HX/XO, while others reported delayed increases in EC [Ca^2+^]_i_ with H_2_O_2_ exposure (Dreher and Junod, 1995; Mcquaid et al., 1996; Okayama et al., 1999).

**FIGURE 3:**
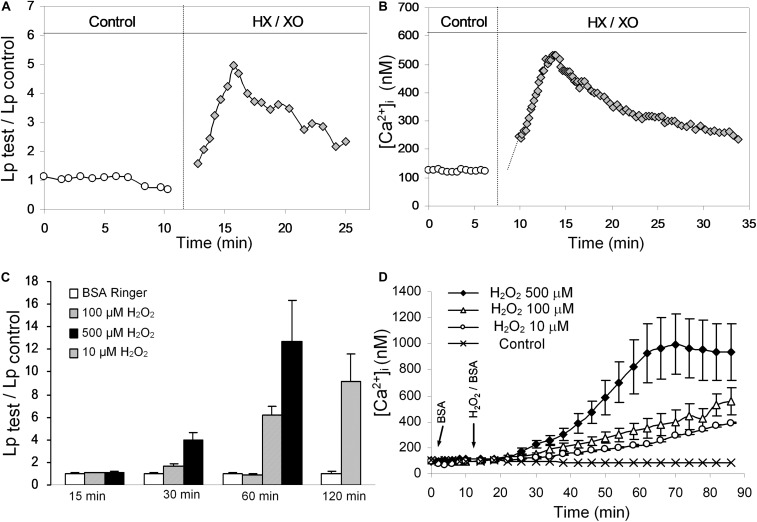
Results of individually perfused rat mesenteric venules demonstrating differential actions of superoxide and H_2_O_2_ on endothelial cell [Ca^2+^]_i_ and microvessel permeability (measured by hydraulic conductivity, Lp). **(A,B)** Individual experiments showing transient increases in Lp and EC [Ca^2+^]_i_ after exposure to superoxide generated by HX/XO. **(C,D)** In contrast to the immediate and transient effects of superoxide, H_2_O_2_ induced delayed and progressive increases in EC [Ca^2+^]_i_ and Lp demonstrating a dose and time-dependent manner. Modified and used with permission (Zhou et al., 2009, 2013).

When the changes in microvessel permeability and endothelial [Ca^2+^]_i_ were assessed under identical experimental conditions in individually perfused intact microvessels, H_2_O_2_ and superoxide demonstrated different effects on EC [Ca^2+^]_i_ and microvessel permeability (Zhou et al., 2009). Figures 3A,B shows that superoxide, a potent reactive oxygen species (generated by HX/XO), induces immediate and transient increases in EC [Ca^2+^]_i_ and microvessel permeability (measured by hydraulic conductivity, Lp) similar to inflammatory mediator-induced responses (Zhou et al., 2009). Whereas H_2_O_2_, a relatively stable reactive oxygen metabolite at a pathologically relevant concentration of 10 μM, induces delayed and progressive increases in EC [Ca^2+^]_i_ and Lp in a time and concentration dependent manner (Figures 3C,D; Zhou et al., 2009, 2013, 2019). Importantly, the increased EC [Ca^2+^]_i_ in either HX/XO or H_2_O_2_ perfused vessels were well correlated with the time course of the increases in microvessel Lp. Inhibiting Ca^2+^ influx by LaCl_3_ prevented the permeability increase induced by HX/XO or H_2_O_2_, thus indicating that Ca^2+^ influx plays an essential role in both superoxide- and H_2_O_2_-induced permeability increases. These findings also indicate that long-term exposure of H_2_O_2_ at a low concentration may cause accumulative lipid oxidation and membrane damage, resulting in Ca^2+^ overload and prolonged irreversible permeability increases (Zhou et al., 2013, 2019). Detailed mechanisms are discussed below. These reported long lasting effects of H_2_O_2_ could play a key role in ROS-mediated vascular dysfunction, which may resemble the pathogenesis of diabetes and other disease associated microvascular dysfunction.

### Methodology Differences in Exogenously Applied ROS

In most of the *in vitro* studies, the applied H_2_O_2_ or HX/XO to generate superoxide was often given to cultured cells as bolus, which causes an immediate reaction of the reactive agents with the cells and other components in the medium and lost the reactive activities quickly. Under those conditions, hundreds of μM or even mM concentrations of peroxide (far more than the pathological concentrations detected in the plasma) were usually needed to see the responses (Alexander et al., 2000; Lee et al., 2006; Jin et al., 2012; Suresh et al., 2015). Under *in vivo* conditions, the effective level of peroxide in the plasma that has direct effect on microvessel endothelial cells is the net amount after the produced ROS in the vasculature were reacted with the plasma antioxidant enzymes. The measured plasma peroxide levels in human with hypertension are close to 10 μM (Lacy et al., 2000) and in diabetic rats are about 10–15 μM (Xia et al., 2018). To better replicate the *in vivo* situations, the studies using individually perfused microvessels, conducted in the absence of blood cell components, directly perfused the vessel with a pathologically relevant net plasma H_2_O_2_ concentration and the changes in EC [Ca^2+^]_i_, microvessel permeability, and cell apoptosis status were assessed while the vessel was continuously exposed to a relatively constant concentration of ROS through the perfusion pipette with constantly refreshed perfusate (Zhou et al., 2013). Comparing to the commonly used bolus application of high concentration of reactive reagents to the tissue or cell culture medium, the continuous perfusion of a pathologically relevant concentration of ROS in intact microvessels closely mimicked the *in vivo* situation. These detailed technical differences may explain some of the differences in results.

### H_2_O_2_-Induced Excessively Produced NO and Microvessel Permeability

The distinct time courses of superoxide and H_2_O_2_-induced increases in microvessel permeability indicate that these two species of ROS may evoke cellular damage via different signaling mechanisms (Zhou et al., 2009, 2013). It is well recognized that low levels of NO participate in a wide range of physiological processes, whereas large amounts of NO lead to nitrosative stress and endothelial dysfunction. Increased production of ROS has been shown to cause increases in microvessel permeability, tissue damage, and organ dysfunction (Del Maestro et al., 1981; Cai and Harrison, 2000; Zhou et al., 2009). A number of studies have suggested that NO regulates the interactions between oxidant-stimulated signaling pathways and endothelial barrier function and protects the cells from ROS-mediated deleterious effects (Clancy et al., 1992; Kubes et al., 1993; Kurose et al., 1995a; Wink et al., 1995; Okayama et al., 1999). For instance, NO has been shown to attenuate oxidant-induced endothelial barrier dysfunction in cultured ECs (Mcquaid et al., 1996; Gupta et al., 2001), in isolated perfused organs (Kavanagh et al., 1994; Poss et al., 1995), and in whole animal studies (Bloomfield et al., 1997; Mcelroy et al., 1997). However, there are also reports where NOS inhibitors do not modulate ROS-induced changes in endothelial barrier function or cell injury (Berman and Martin, 1993; Az-Ma et al., 1996) or even NO can exacerbate H_2_O_2_-induced endothelial permeability in cultured ECs (Mcquaid et al., 1996; Okayama et al., 1997). It is thus apparent that the roles of endogenous vs. exogenous or basal vs. excessive NO in ROS-mediated endothelial barrier function remain equivocal and warrants more evidence to determine the conditions where NO may act either as a proinflammatory or protective mediator *in vivo*.

**FIGURE 4:**
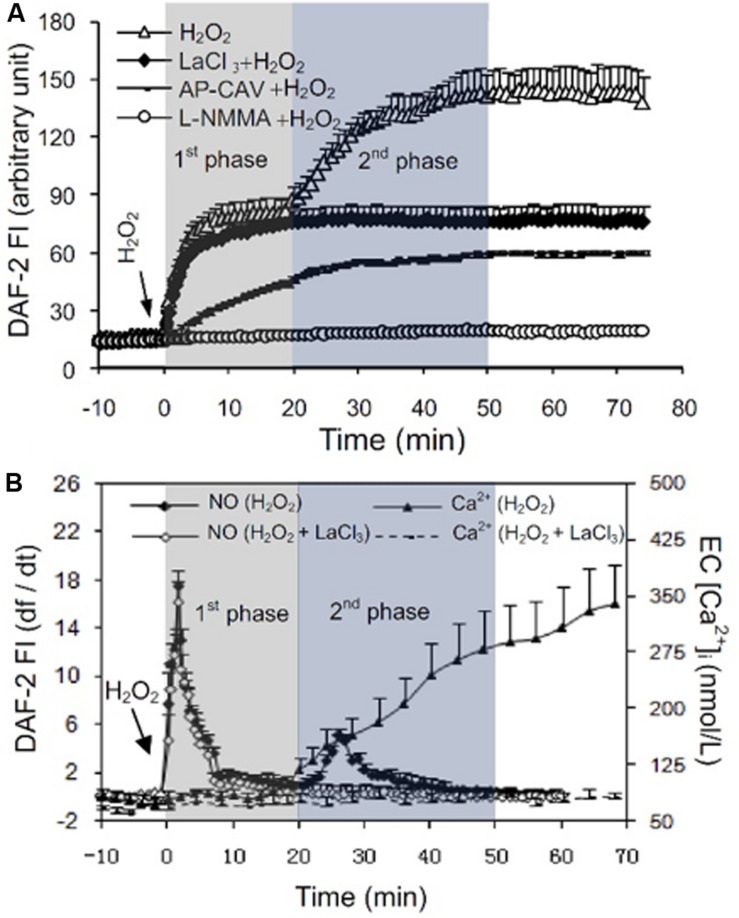
Measurements of H_2_O_2_-induced EC NO in DAF-2 DA loaded rat mesenteric venules. **(A)** Perfusion of vessels with H_2_O_2_ (10 μmol/L) increases EC NO via an initial Ca^2+^ influx-independent (1st) phase followed by a 2nd Ca^2+^-dependent phase. The application of NOS inhibitors *N*^G^-monomethyl-L-arginine (L-NMMA) or AP-CAV prevent or reduce H_2_O_2_-induced NO in both phases, and blocking Ca^2+^ influx with LaCl_3_ only prevented the 2nd phase of NO production. **(B)** Superimposed time courses of NO production rate (df/dt, calculated from the cumulative DAF FI curve, left *Y*-axis) with the changes in EC [Ca^2+^]_i_ (right *Y*-axis) in H_2_O_2_-perfused vessels demonstrate that the H_2_O_2_-induced increases in EC [Ca^2+^]_i_ only started to increase during the 2nd phase of increased NO, but increased progressively thereafter. Used with permission (Zhou et al., 2013).

**FIGURE 5:**
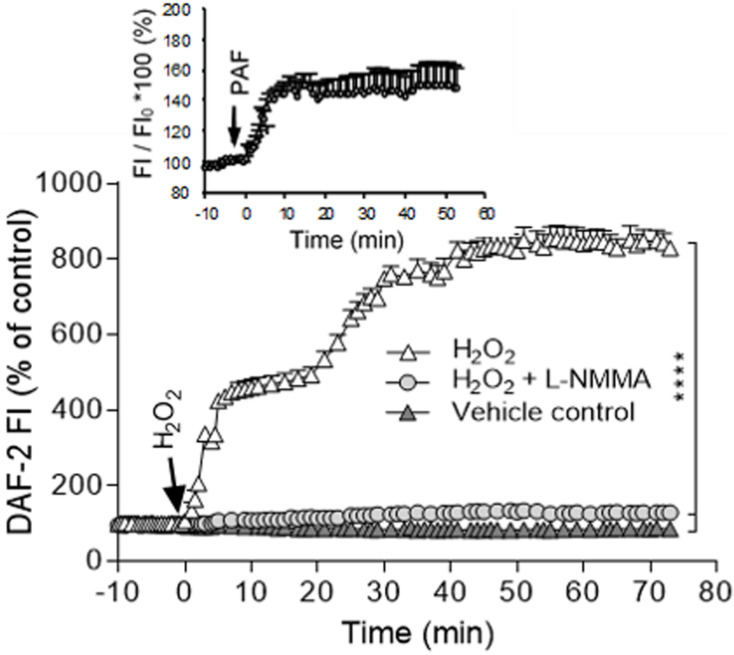
Comparison of PAF and H_2_O_2_-induced NO production in rat mesenteric microvessels. NO was measured in DAF-2 DA loaded microvessels. When the vessel was exposed to PAF, the cumulative change of DAF-2 FI at plateau level was about 1.5 times of that of the control and lasted about 6 min (plateau indicates no further increased NO production). Whereas in H_2_O_2_ (10 μM) perfused microvessels, the maximum change of DAF-2 FI was about nine times of that of the control, about six times higher than that of PAF-induced NO production. Importantly, the increased NO lasted about 50 min. Modified and used with permission (Zhu and He, 2005; Zhou et al., 2019).

**FIGURE 6:**
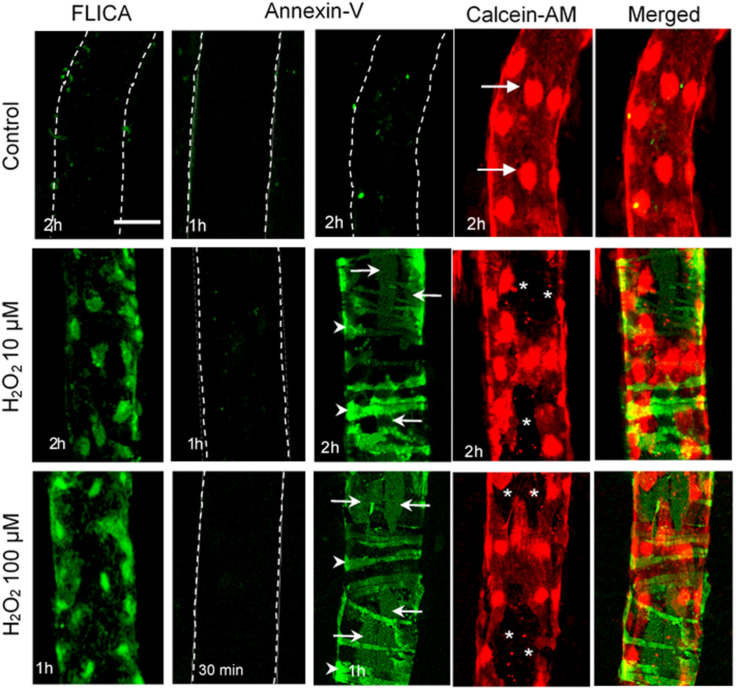
H_2_O_2_-induced vascular cell apoptosis. Representative confocal images of FLICA (indicator for activated caspase 3/7), Alexa488-Annexin-V (labeling externalized phosphatidylserine, PS), Calcein-AM (live cell staining), and merged images of Annexin-V with Calcein-AM in H_2_O_2_-perfused rat mesenteric venules. These images illustrate that H_2_O_2_ induces time and dose dependent caspase activation and vascular cell apoptosis. Importantly, pericytes start apoptosis earlier than endothelial cells (ECs) in the same vessel segment (i.e., with identical H_2_O_2_ concentration and exposure time), suggesting their higher vulnerability to ROS than ECs. Arrows indicate ECs and arrowheads indicate pericytes. *ECs not stained with Calcein-AM. Used with permission (Zhou et al., 2013).

H_2_O_2_-induced increases in EC NO production have been indicated to play distinct roles in different vasculature (Cai, 2005a). H_2_O_2_ is considered an important vasodilator in large arteries or arterioles through its activation on eNOS and increased NO production (Yang et al., 1999; Cai et al., 2003). Whereas in venules, the H_2_O_2_-induced excessive NO was linked to nitrosative stress and endothelial dysfunction (Yuan et al., 1993; Baluk et al., 1997; Zhu and He, 2005; Hatakeyama et al., 2006; Zhou and He, 2010, 2011; Mundi et al., 2017; Zhou et al., 2019). H_2_O_2_-induced increases in EC NO and the relationship between NO and EC [Ca^2+^]_i_ were characterized in detail from studies conducted in intact microvessels and presented in Figure 4 (Zhou et al., 2013). It was demonstrated that H_2_O_2_ at 10 μM, a concentration close to the plasma level in human disease conditions (Lacy et al., 2000), induced an immediate and long lasting increased NO production, which was characterized by two phases. The initial phase with highest NO production rate occurred immediately upon H_2_O_2_ application, which was correlated with increased eNOS Ser^1177^ phosphorylation in the absence of elevated [Ca^2+^]_i_ and did not cause a corresponding increase in permeability. The second phase of increased NO occurred after 20 min of H_2_O_2_ perfusion when EC [Ca^2+^]_i_ started to increase, and was associated with Ca^2+^-dependent Thr^495^ dephosphorylation and blocked by LaCl_3_, a blocker for Ca^2+^ influx (Figure 4; Zhou et al., 2013). Comparing platelet activating factor (PAF)-induced NO production that occurred downstream from transient increases in EC [Ca^2+^]_i_ and was required for increases in microvessel permeability, the magnitude of H_2_O_2_-induced NO was about 6 times of PAF-induced NO (Figure 5). Most importantly, H_2_O_2_-induced NO lasted 50 min, much longer than that of PAF-induced NO (6 min). This large amount of NO, instead of causing an immediate increase in microvessel permeability that occurred in PAF-stimulated microvessels (Zhu and He, 2005; Zhou and He, 2010), lead to caspase activation, delayed and cumulative increases in EC [Ca^2+^]_i_, vascular cell apoptosis, and progressively increased microvessel permeability (Figures 3, 6). The results also showed that the H_2_O_2_-induced caspase activation not only results in increases in EC [Ca^2+^]_i,_ but the increased [Ca^2+^]_i_ can further activate caspases. Blockade of Ca^2+^ influx by LaCl_3_ significantly attenuated H_2_O_2_-induced caspase activation. These observations suggested that H_2_O_2_-induced NO-mediated [Ca^2+^]_i_ overload and cell apoptosis can be augmented through a positive feedback mechanism. Importantly, inhibition of NOS prevented all of the H_2_O_2_-induced events including caspase activation, cell apoptosis, increases in endothelial [Ca^2+^]_i_ and microvessel permeability, indicating a crucial role of NO in H_2_O_2_-mediated vascular barrier dysfunction. However, the H_2_O_2_-induced largely increased NO (1st phase), in the absence of increased EC [Ca^2+^]_i_, did not increase microvessel permeability, suggesting excessive NO alone is not sufficient to increase microvessel permeability. By linking the timing of H_2_O_2_-induced increases in microvessel Lp (Figure 3C) with the superimposed time courses of H_2_O_2_-induced changes in EC NO and [Ca^2+^]_i_ shown in Figure 4B, the Lp increase only occurred after EC [Ca^2+^]_i_ was significantly elevated. Therefore, these studies evidently indicate that it is NO-mediated EC [Ca^2+^]_i_ overload that causes increases in permeability. The mechanisms involved in the excessive NO-mediated impairment of EC Ca^2+^ homeostasis are discussed in the flowing section with the focus on the interplay between ROS, NO, and reactive nitrogen species in vascular barrier dysfunction.

### ROS, NO, ONOO^–^, and Microvessel Permeability

Increased production of ONOO^–^ has been implicated to be associated with inflammation and various cardiovascular diseases (Eschwege et al., 1999; Knepler et al., 2001; Wu and Wilson, 2009). ONOO^–^, a highly reactive oxidant produced by the reaction of superoxide with NO, is capable of oxidizing lipid membranes, nucleic acids, and metabolic enzymes (Beckman et al., 1990; Beckman and Koppenol, 1996). A compelling body of evidence supports a role of ONOO^–^-mediated damage at inflammatory site (Salvemini et al., 1998). ONOO^–^ has been reported to cause disruption of endothelial cytoskeletal proteins (Knepler et al., 2001; Neumann et al., 2006) or activate upstream signaling cascades (Vepa et al., 1997), leading to EC barrier dysfunction. In cultured ECs, ONOO^–^ has been shown to increase basal and agonist-stimulated Akt- and AMP-activated Ser^1179^ phosphorylation of eNOS, but decreased NO production and bioactivity (Zou et al., 2002). In addition, while ONOO^–^-induced accumulation of cGMP in ECs could regulate EC shape and function, activation of poly(ADP-ribose) synthetase (PARS) by ONOO^–^ is reported to suppress mitochondrial respiration and cause endothelial dysfunction (Szabo et al., 1997). These findings suggest that ONOO^–^ can potentially alter endothelial function through complex mechanisms.

**FIGURE 7:**
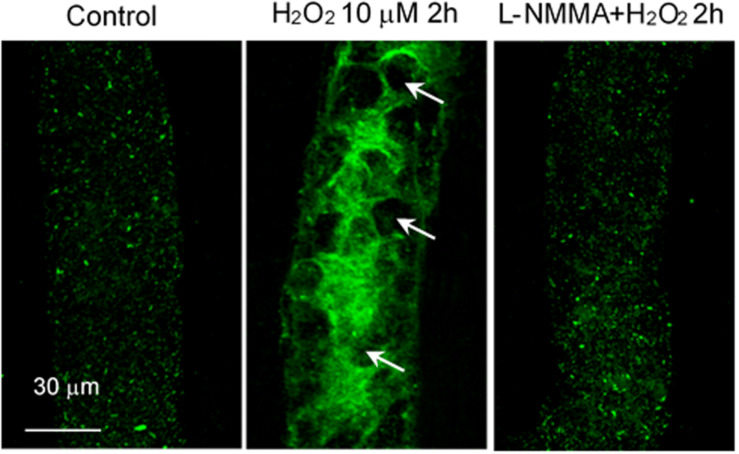
H_2_O_2_ induced NO-dependent tyrosine nitration in rat mesenteric venules. Confocal images showing the fluorescent immunostaining of nitrotyrosine in vessels perfused with control (Ringer solution containing 1% BSA, left), H_2_O_2_ (10 μmol/L, middle, arrows indicate the nucleus of ECs without nitrated tyrosine formation), and H_2_O_2_ in the presence of L-NMMA (2 mmol/L, right). Used with permission (Zhou et al., 2019).

**FIGURE 8:**
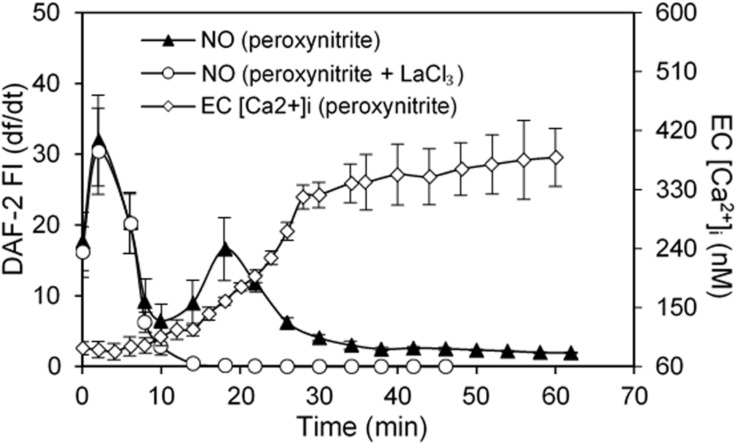
ONOO^–^ induced NO production and increases in EC [Ca^2+^]_i_ in perfused rat mesenteric venules. Perfusion of vessels with ONOO^–^ (50 μM) increased NO in 2 phases followed by increases in EC [Ca^2+^]_i_. The NO production rate (df/dt, left *Y* axis) derived from cumulative DAF-2 FI in ONOO^–^-perfused vessels is superimposed with the time course of increased EC [Ca^2+^]_i_ (right *Y* axis). The application of LaCl_3_ (50 μM) did not affect ONOO^–^-induced initial NO production but prevented the 2nd phase of NO production, indicating the 2nd phase of NO is Ca^2+^influx-dependent. Importantly, EC [Ca^2+^]_i_ only started to increase after 20 min of ONOO^–^ perfusion, but increased progressively thereafter, a pattern similar to that observed in H_2_O_2_-perfused vessels. Used with permission (Zhou et al., 2019).

**FIGURE 9:**
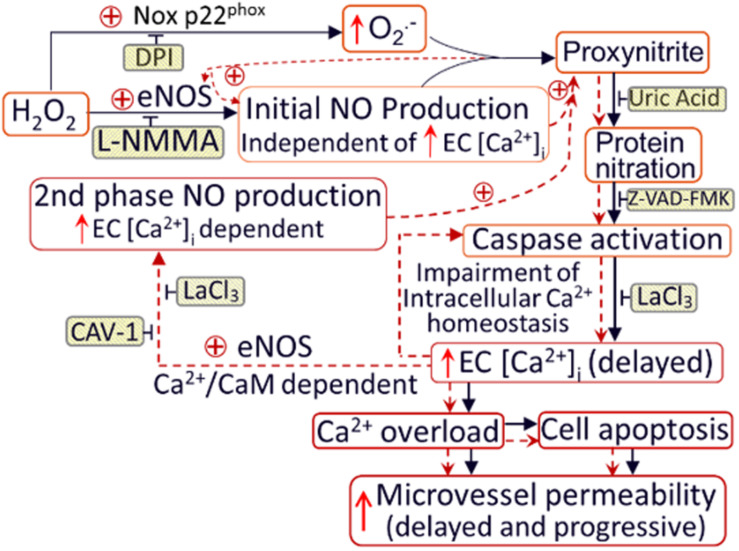
Schematic diagram illustrating the mechanisms of H_2_O_2_-induced microvessel barrier dysfunction. H_2_O_2_ at pathophysiological levels activate eNOS and NADPH oxidase in the endothelium and pericytes, leading to increased formation of NO and O_2_^–^. The temporal and spatial proximity of largely produced NO and O2^–^ promote the formation of ONOO^–^, a more potent oxidant, leading to ONOO^–^-mediated lipid peroxidation, protein tyrosine nitration, subsequent impairment of intracellular Ca^2+^ homeostasis, and vascular cell apoptosis. Both NO-derived ONOO^–^ and the delayed increases in EC [Ca^2+^]_i_ are able to further activate eNOS, resulting in amplified NO production and NO-dependent downstream cascade. These positive feedback loops, indicated by red dotted lines, play an important role in sustained production of ONOO^–^, and eventually ONOO^–^-mediated endothelial cell Ca^2+^ overload, vascular cell apoptosis, and progressively increased microvessel permeability. The application of inhibitors (shaded boxes) blocks the downstream events. DPI (diphenylene iodonium, NADPH oxidase inhibitor); eNOS: endothelial nitric oxide synthase; CAV-1: Caveolin-1 (eNOS inhibitor); z-VAD-FMK: caspase inhibitor. Used with permission (Zhou et al., 2019).

H_2_O_2_, in addition to inducing NO production, also increases superoxide production, which has been considered as important feed-forward mechanisms for ROS-mediated pathogenesis of cardiovascular diseases (Cai, 2005b; Zinkevich and Gutterman, 2011). *In vitro* studies reported that the H_2_O_2_-induced superoxide production was prevented by the inhibition of p22^phox^ with siRNA and knockout of gp91^phox^, the two transmembrane subunits of NAD(P)H oxidase, in cultured vascular endothelial cells (Li et al., 2001; Djordjevic et al., 2005). To further elucidate the underlying mechanism for H_2_O_2_-induced NO-dependent delayed increases in EC [Ca^2+^]_i_, cell apoptosis, and progressively increased microvessel permeability, additional studies were conducted in intact microvessels to explore the potential involvement of NO-derived other form of oxidant agent (Zhou et al., 2019). Consistent with others report, the study first demonstrated increased production of superoxide in both ECs and pericytes in H_2_O_2_ perfused vessels, which was inhibited by DPI, a commonly used NAD(P)H oxidase inhibitor, supporting NAD(P)H oxidase as the primary source for the H_2_O_2_-induced superoxide production (Zhou et al., 2019). This increased superoxide was not only concurrent but also in close proximity with H_2_O_2_-induced markedly increased NO, thus resulting in spontaneous generation of ONOO^–^. The direct evidence of H_2_O_2_-induced ONOO^–^ formation is demonstrated with an extensive NO-dependent tyrosine nitration in H_2_O_2_ perfusion microvessels (Figure 7). Nitrotyrosine is a well-recognized biomarker for ONOO^–^, which commonly served as an indication of systemic nitroxidative stress (Radi, 2013; Zhou et al., 2019). The direct effects of ONOO^–^ in intact microvessels and its role in H_2_O_2_-induced permeability increase were further characterized in a recent study conducted in intact microvessels. The study showed that the NO-derived ONOO^–^ plays an important role in H_2_O_2_-induced increases in microvessel permeability and the application of an endogenous ONOO^–^ scavenger, uric acid, prevents H_2_O_2_-induced increases in endothelial [Ca^2+^]_i_ and microvessel permeability. The application of exogenous ONOO^–^ in microvessels also showed delayed dose- and time-dependent increases in EC [Ca^2+^]_i_ and permeability, a pattern similar to that induced by H_2_O_2_ (Figure 8). Most importantly, exogenous ONOO^–^ activates eNOS and further increases NO production, resulting in amplified ONOO^–^ formation and severe tissue damage. These findings suggest that H_2_O_2_-induced excessive NO-derived ONOO^–^ is the primary mediator of H_2_O_2_-induced cell injury and progressively increased microvascular permeability. The fact that ONOO^–^ could cause further activation of eNOS with amplified NO constitutes a self-promoted augmentation of nitroxidative stress, which results in progressively increased permeability. The signaling cascade and the sequential events found in H_2_O_2_-perfused intact microvessel are illustrated in Figure 9 (Zhou et al., 2019), which provide mechanistic insights into a better understanding of the pathogenesis of oxidant-induced RNS-mediated microvascular dysfunction.

## Role of Basal No in Microvessel Barrier Function

Basal NO and agonist-induced excessive NO have been shown to play different roles in the regulation of microvessel permeability (Zhu and He, 2005; Zhou and He, 2010; Xu et al., 2013). As discussed above, ROS- or inflammatory mediator-induced excessive NO contributes to increased vascular permeability through different mechanisms (Moncada et al., 1991; Yuan et al., 1993; Mayhan, 1994; Wu et al., 1996; He et al., 1997; Zhu and He, 2005; Hatakeyama et al., 2006; Sanchez et al., 2009; Zhou and He, 2011), whereas basal NO was reported to play a critical role in preventing leukocyte adhesion (Furchgott and Vanhoutte, 1989; Kubes et al., 1991; Moncada et al., 1991) and the adhesion and aggregation of platelets (Radomski et al., 1990a, b). Reduced basal NO synthesis or NO bioavailability has been reported in various disease conditions including hypertension, diabetes, hypercholesterolemia, and reperfusion injury, which was commonly linked to endothelial dysfunction and vascular inflammation (Moncada and Higgs, 2006; Potenza et al., 2009). Genetic deletion of eNOS or over expression of caveolin-1, an endogenous inhibitor of eNOS, has been shown to cause increased leukocyte adhesion and macrophage infiltration, and accelerated atherosclerosis in apolipoprotein E (apoE) deficient mice (Sasaki et al., 2003; Fernandez-Hernando et al., 2009; Atochin and Huang, 2010; Fritzsche et al., 2010; Ponnuswamy et al., 2012). Those studies indicated an important role of basal NO in maintaining vascular function and preventing vascular inflammation, but the mechanisms were not well defined. Although the animals with genetic deletion of eNOS generated a constant reduction of NO systemically, such condition may trigger complex adaptation and compensatory effects in the vascular system. Therefore, the inflammatory manifestation observed in those animals may not unequivocally represent the role of basal NO.

**FIGURE 10:**
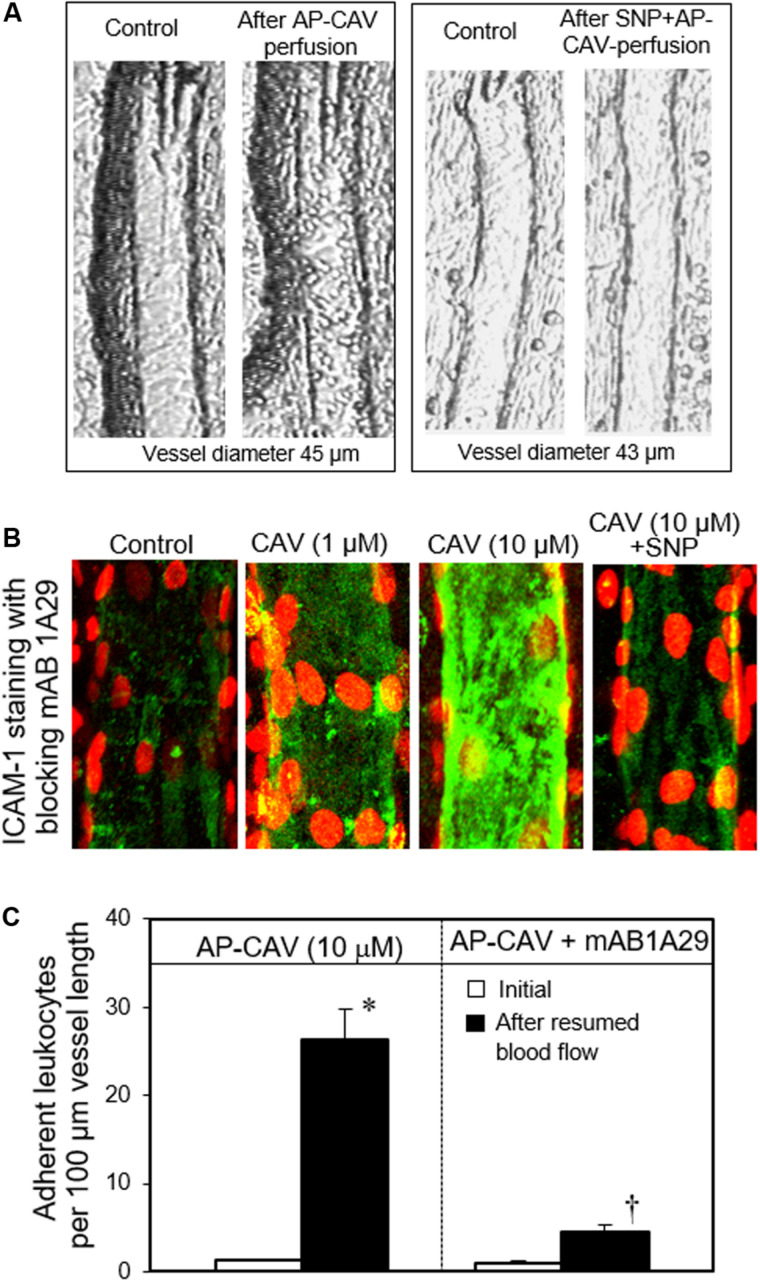
Perfusion of rat mesenteric venules with AP-CAV, an endogenous NOS inhibitor, induced basal NO-dependent ICAM-1 mediated leukocyte adhesion. Intact venules were perfused by AP-CAV for 30 min followed by resuming blood flow in the same vessel for 10 min. Leukocyte adhesion was quantified when each vessel was recannulated with BSA-Ringer solution. **(A)** Video images of a perfused venule under control conditions and after AP-CAV (10 μM)-induced leukocyte adhesion, and the administration of a NO donor, sodium nitroprusside (SNP), abolished AP-CAV-induced leukocyte adhesion. **(B)** AP-CAV induced dose-dependent increases in the binding affinity between EC ICAM-1 and its blocking antibody mAb1A29. Confocal images of mAb1A29 (green) and vascular cell nuclei (red) immunofluorescence co-staining under control conditions, after AP-CAV perfusion, and after adding SNP to AP-CAV perfused vessels. **(C)** Perfusion of vessels with ICAM-1 inhibitory antibody, mAb1A29, significantly attenuated AP-CAV induced leukocyte adhesion. * and † indicate a significant increase and decrease from the control, respectively. Used with permission (Xu et al., 2013).

Some *in vivo* studies demonstrated that the application of NOS inhibitor through either systemic injection or superfusion to the vascular beds induced increases in microvessel permeability (Kubes and Granger, 1992; Kurose et al., 1995b). However, reduced basal NO in the presence of blood would cause leukocyte and platelet adhesion, which may confound the direct effect of basal NO on permeability. When studies were conducted in individually perfused vessels in the absence of blood components in the vessel lumen, reduced basal NO production by NOS inhibitor or caveolin-1 peptide, AP-CAV, showed no effect on basal microvessel permeability (Xu et al., 2013). The increased permeability observed in whole vascular bed studies could be attributed to the reduced basal NO-induced increases in adherent leukocytes. In the presence of any inflammatory stimulus, the adherent leukocytes could be activated to release ROS and thus increase the permeability. The mechanisms of reduced basal NO-induced leukocyte adhesion were demonstrated recently (Xu et al., 2013; Gao et al., 2017). Those studies showed that when blood flow was resumed in vessels that were pretreated with NOS inhibitor or AP-CAV to reduce endothelial basal NO, large amounts of leukocytes quickly adhered to rat venules and the adhesion was demonstrated to be EC ICAM-1 mediated (Figure 10; Xu et al., 2013). Most importantly, the ICAM-1-mediated adhesion occurred very quickly (less than 30 min), which rules out the possibility of *de novo* protein synthesis and translocation. Such rapid changes in ICAM-1 adhesiveness predicts a NO-dependent regulation of the adhesive binding avidity of constitutively expressed endothelial ICAM-1. This prediction was further validated by an additional *in vitro* cell culture study showing that the reduced basal NO-induced increases in EC adhesive binding were mediated by Src-dependent ICAM-1 activation through its phosphorylated state (Gao et al., 2017).

Other *in vitro* studies proposed that the inhibition of eNOS-derived NO does not directly contribute to EC conversion to a pro-adhesive phenotype but rather interferes with the generation of ROS by NADPH oxidase and results ROS-mediated inflammatory response (Niu et al., 1994; Kvietys and Granger, 2012). In contrast to this proposal, the timing and pattern of leukocyte adhesion observed in intact microvessels (Xu et al., 2013) strongly support a direct association between basal NO and adhesive states of ECs. Importantly, if the increased adhesiveness of ECs is ROS mediated, an increased permeability should be observed. Instead, the results showed that basal NO reduction-induced adherent leukocytes do not cause permeability increase unless additional stimuli (such as fMLP) are applied to trigger neutrophil respiratory burst (Xu et al., 2013). Taken together, these studies demonstrated that basal NO is essential for maintaining the non-adhesive state of endothelium through regulation of the adhesive binding of endothelial ICAM-1.

## Role of ROS in Pericyte Loss and Microvessel Permeability

The albuminal side of endothelium is surrounded by a morphologically distinct cell termed pericyte. Pericyte was first described as perivascular contractile cell that wrap around the ECs of microvessels (Sims, 1986). They are also known as Rouget cells named after their discoverer, or referred to as mural cells because of their perivascular position, or as microvascular smooth muscle cells (SMCs) because of their contractile nature (Armulik et al., 2011; Stapor et al., 2014; van Dijk et al., 2015; Ferland-McCollough et al., 2017).

### Pericyte Structure and Function in Microvessels

Pericytes are found on pre-capillary arterioles, capillaries, post-capillary venules, and collecting venules of many organs, but they are more extensive in postcapillary values (Sims, 1986; Shepro and Morel, 1993). They are embedded within the endothelial basement membrane (BM) and have a cell body with a prominent nucleus, a small amount of cytoplasm with extending long processes along the longitudinal axis of the blood vessels, and usually span many ECs (Armulik et al., 2011). The morphology of pericyte is also reported to be very distinct in different organs ranging from a typical flattened or elongated, stellate-shaped cell with multiple cytoplasmic processes encircling a large albuminal vessel area in central nervous system (CNS) to that of a mesangial cell of kidney glomerulus as rounded, compact, and covering a minimal albuminal vessel area (Armulik et al., 2005; Hartmann et al., 2015). The pericyte coverage of the endothelial albuminal surface varies at different organs and vascular beds, and it reflects the relative ratio between the ECs to pericytes (Sims, 1986). The highest pericyte coverage of microvessels is reported in CNS and retina with EC-to-pericyte ratio of 1:1 to 3:1 forming blood-brain barrier and blood-retinal barrier, and the lower EC-to-pericyte ratio of 100:1 in skeletal muscle (Sims, 1986; Shepro and Morel, 1993). Pericytes and ECs not only share a common BM, they are directly connected to each other through the interruptions in the BM (Rucker et al., 2000). The intimate relationship between albuminal pericytes and luminal ECs through the BM is not only the physical communications; it involves several signaling mechanisms that regulate pericyte-EC interactions and communication. The signaling pathways have been extensively reviewed by several authors (Armulik et al., 2005; von Tell et al., 2006; Gaengel et al., 2009; Armulik et al., 2011; Hill et al., 2014; Warmke et al., 2016). Investigations with genetic mouse models have showed that pericytes and ECs are interdependent, as a result a primary defect in one cell type have inevitable consequences on the other cell (Armulik et al., 2005; Gaengel et al., 2009). Because of its intimate relationship with endothelium and BM, pericytes have recently come into focus as an emerging key factor in the regulation of diverse vascular functions both in health and disease. Briefly, pericytes are functionally associated with the regulation of vascular contraction and blood flow (Shepro and Morel, 1993; Rucker et al., 2000; Bergers and Song, 2005; van Dijk et al., 2015), vessel permeability (Edelman et al., 2006; Yuan and He, 2012), vascular stability (von Tell et al., 2006), BM organization (Stratman and Davis, 2012), and angiogenesis (Gerhardt and Betsholtz, 2003; Bergers and Song, 2005).

### Role of Pericytes in the Regulation of Microvessel Permeability

**FIGURE 11:**
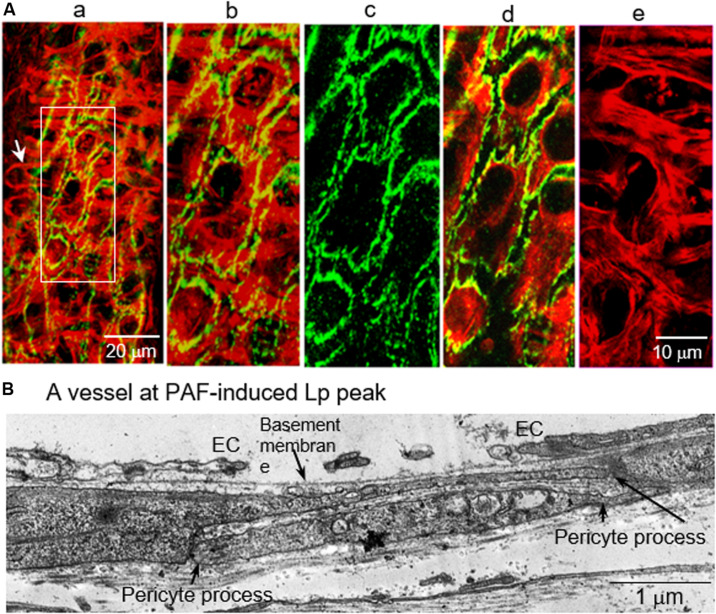
Confocal and electron micrographs of rat mesenteric venules demonstrating the positioning of pericyte processes in relation to open endothelial junctions at PAF-induced peak increase of Lp. **(A)** Confocal images: Image a is a partial projection of the lower half of the vessel wall with dual staining of F-actin (red) and VE-cadherin (green). An arrow indicates a typical pericyte structure: a single nucleus with multiple cellular processes. Details of a local region of the vessel wall (rectangular box) are displayed in images b to e with a higher magnification. Image b is a dual channel image projection of both endothelial and pericyte layers demonstrating that all of the open VE-cadherin is covered by aggregated pericyte actin bundles. Image c shows large separations of endothelial VE-cadherin. Image d is a dual channel image projection at the endothelial cell layer showing the changes in endothelial F-actin and VE-cadherin. Image e shows the aggregated pericyte F-actin bundles (a projection of image sections blow endothelial cells). Micrograph **(B)** shows the structural changes in vascular wall at the PAF-induced Lp peak. It displays a wide open endothelial gap with intact basement membrane. Consistent with confocal images, two extended overlapping pericyte processes provide a complete coverage of the endothelial gap. These images suggest a role of pericytes in stabilizing the vascular wall and lessening an otherwise greater magnitude of leakage while endothelial barrier is impaired. Modified and used with permission (Yuan and He, 2012).

Vascular endothelial cells have been recognized as the principal barrier for fluid and solute exchange between circulating blood and surrounding tissues. During inflammation, the increased permeability mainly occurs at venules. The majority of the venular wall is wrapped by perivascular cells, mainly pericytes (Murfee et al., 2005). The prime location of pericytes suggests that they may serve as an additional barrier at the vessel walls that restricts macromolecule leakage when permeability is increased. Additionally, based on its contractile nature, it may also actively involve the regulation of microvascular permeability. The role of pericytes in microvessel barrier function has been controversial. Some of the studies reported that the contraction of pericytes prompted the gap formation between endothelial cells (Kelley et al., 1988). While other *in vivo* studies indicated that pericytes might protect endothelial junctions upon histamine exposure (Sims et al., 1990). In a microvascular lung pericyte/EC co-culture study, addition of pericytes is reported to increase the permeability barrier compared to EC alone (Dente et al., 2001). A study using a pericyte deficient mouse model revealed that the lack of pericytes increased the permeability of BBB to water and a range of solute tracers (Armulik et al., 2010; Daneman et al., 2010). Another *in vivo* evidence derived from inflamed vessels demonstrated that pericyte F-actin bundles and pericyte processes completely covered largely separated VE-cadherin and open endothelial gaps at the peak of the permeability increases (Yuan and He, 2012). Figure 11 illustrated details with confocal images and ultrastructural micrograph. These *in vivo* observations suggest that pericytes, instead of promoting endothelial gap formation, play an important role in lessening a potentially higher degree of leakage when endothelial barrier is impaired. In vessels going through inflammation-induced vascular remodeling, the EM structure of the vascular walls showed proliferated perivascular cells and expanded extracellular matrix, which not only serve as a secondary barrier that lessen the vascular leakage but also provide a supporting structure to stabilize the microvessel wall during stimulation-induced EC cytoskeleton contractions (Yuan and He, 2012).

### ROS-Mediated Pericyte Apoptosis

**FIGURE 12:**
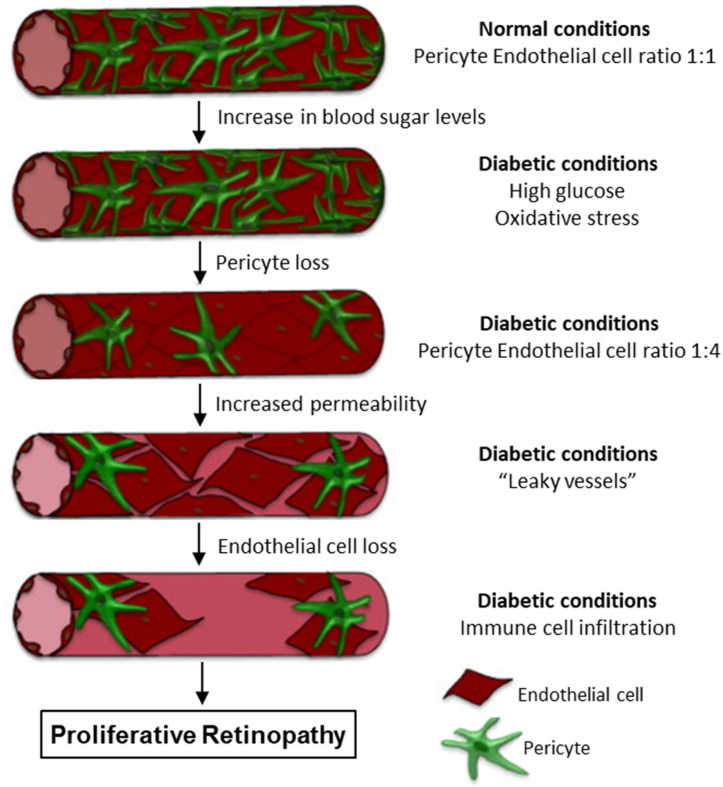
Retina vascular progression to proliferative retinopathy under diabetic conditions. Hyperglycemia and oxidative stress cause pericyte apoptosis and increases in microvessel permeability. The loss of pericytes and endothelial cells will further increase fluid and protein leakage in the retina and immune cell infiltration. These intra-ocular vascular changes will then contribute to the development of proliferative retinopathy. Modified and used with permission (Ferland-McCollough et al., 2017).

As pericytes are abundant in microvasculature and have been linked to a wide array of vascular functions, alterations in pericyte coverage or pericyte dysfunctions can contribute to multiple vascular pathologies. Accordingly, pericyte loss has been demonstrated in the pathogenesis of several microvascular complications under disease conditions (Armulik et al., 2005; Hill et al., 2014; van Dijk et al., 2015; Warmke et al., 2016; Ferland-McCollough et al., 2017). The most characterized and established microvascular complications with pericyte loss are associated with diabetes, such as diabetic retinopathy, diabetic nephropathy, and diabetic neuropathy (van Dijk et al., 2015; Warmke et al., 2016; Ferland-McCollough et al., 2017). Figure 12 illustrates the progression of retina vessels from hyperglycemia and oxidative stress-induced loss of pericytes and endothelial cells, increased vascular permeability, to the development of proliferative retinopathy (Ferland-McCollough et al., 2017). Loss of pericyte number and coverage has also been reported in neurodegeneration and Alzheimer’s disease (Sagare et al., 2013; Sengillo et al., 2013). Ischemic stroke within CNS is reported to cause microvascular pericyte loss during ischemia/reperfusion (Hill et al., 2014). However, the mechanisms involved in pericyte loss under those pathological conditions remain unclear. In all of the diseases mentioned above, oxidative stress plays an important role and ROS-induced cell injury could be the main cause leading to cell apoptosis. An early indicator of apoptosis is the translocation of phosphatidylserine (PS) from the inner to the outer leaflet of the plasma membrane, which can be detected by Annexin V, a phospholipid-binding protein that has high affinity for PS. As that shown in Figures 3, 6, perfusion of microvessels with H_2_O_2_ at 10 μM for 2 h caused caspase activation, cell apoptosis, followed by EC Ca^2+^ overload and increased microvessel permeability (Zhou et al., 2013). Importantly, when the apoptotic cells in H_2_O_2_-perfused vessels were illustrated by Annxin-V staining (Figure 6), pericytes undergo apoptosis earlier than most of the ECs, albeit both of them were exposed to the same concentration of H_2_O_2_ with identical exposure time. This observation indicates that pericyte are more vulnerable to oxidative stress than ECs. This may also explain the early loss of microvascular pericytes that was commonly found in diabetic patients and animals as that shown in Figure 12. The loss of pericytes in retina microvessels could be the main cause for aneurism formation in diabetic retinopathy and the increased plasma levels of ROS could play an important role (Shojaee et al., 1999).

In summary, in recent years our knowledge in pericyte characterization, functions, and its involvement in pathological conditions has evolved significantly, but there are far more questions than answers. The multifaceted role of pericytes thus emphasizes the need of further studies in intact microvasculature with novel *in vivo* and *ex vivo* experimental approaches to fully understand the specific pericyte function in health and disease, and to further explore their regenerative potential and the signaling pathways affected vascular function in different pathological states.

## Oxidative Stress and Antioxidant Counter Balance

It is now well recognized that the state of oxidative stress in the vascular wall is developed following an imbalance between ROS and antioxidant enzymes where excess cellular production of ROS outperforms the capacity of endogenous antioxidant defense mechanisms. Growing evidence from basic and clinical studies suggest that increased oxidative stress plays a pivotal role in the initiation and progression of many cardiovascular diseases (Madamanchi et al., 2005; Landmesser et al., 2007; Forstermann, 2008; Boueiz and Hassoun, 2009). While animal studies supported the potential role of antioxidants in preventing oxidative stress-induced vascular disorders (Modig and Sandin, 1988; Bernard, 1991; Leff et al., 1993), clinical trials with various antioxidants have been disappointing and failed to demonstrate conclusive results (Madamanchi et al., 2005; Landmesser et al., 2007; Forstermann, 2008; Boueiz and Hassoun, 2009). While several factors have been identified for the lack of clinical efficacy of antioxidants (Madamanchi et al., 2005; Forstermann, 2008; Boueiz and Hassoun, 2009), it has become essential to have a better understanding of ROS-mediated signaling mechanisms, their localization, and the integration of both ROS-dependent transcriptional and signaling pathways in vascular pathophysiology (Madamanchi et al., 2005). Consequently, the field of oxidative stress has recently been widened significantly and it is often viewed as an imbalance of genes encoding antioxidant enzymes and proteins, and how those genes are regulated under disease conditions with increased ROS production.

Antioxidant enzymes such as SOD, catalase, and glutathione peroxidase are able to break down ROS promptly to other less reactive and non-reactive downstream products. These proteins play key roles in counter balance of oxidative stress-induced cell damage. The regulation of cellular redox homeostasis mainly occurs at the transcription level. Cells activate transcription of protective antioxidant genes under oxidative stress via varieties of redox-sensitive transcription factors, of which Nrf2 has been recognized as a master regulator of antioxidative responses (Motohashi and Yamamoto, 2004; Amin et al., 2019). Under normal physiological conditions, Nrf2 is located in the cytosol at a low concentration and is the target for proteasomal degradation by its interaction with a cytosolic regulatory protein Kelch-like ECH-associated protein (Keap1) (Nguyen et al., 2009). With increased ROS, Nrf2 dissociates from Keap1 and translocates to the nucleus where it binds to the promoters of genes containing the *cis*-acting antioxidant response element (ARE). Its binding to ARE at nucleus promotes the transcription of a wide variety of antioxidant genes (Ma, 2013). Many ARE genes are involved in detoxification enzymes such as glutathione-S-transferases, heme oxygenase-1, and NAD(P)H dehydrogenase (Nguyen et al., 2009) and hence the Nrf2 activity has been viewed as a key indicator of cellular antioxidant capacity. Since many factors may affect Nrf2 activity such as its dissociation from Keap1, translocation to nucleus, its efficiency in binding to ARE due to enhanced nuclear export and degradation, exactly how Nrf2 activity is regulated under different pathological condition-induced oxidative stress remains to be uncovered. The Nrf2/Keap1-ARE signaling pathways and biochemical aspect of their interactions under different disease conditions have been reviewed elsewhere (Chen et al., 2015; Amin et al., 2019).

Nrf2 dysfunction or reduced Nrf2 activity has been found in certain oxidative stress-associated conditions such as aging-related vascular inflammation (Usatyuk and Natarajan, 2004; Ungvari et al., 2011b), obesity-induced neurovascular dysfunction (Tarantini et al., 2018), and the disturbed flow-mediated atheroprone regions of the vasculature (Mcsweeney et al., 2016). Genetic deletions of Nrf2 in mice and rats are viable under normal conditions, but they have shown higher sensitivity to increased oxidant stress-induced vascular damages and organ dysfunction (Chan and Kwong, 2000; Chan et al., 2001; Chanas et al., 2002; Ungvari et al., 2011a; Priestley et al., 2016). High fat diet-induced increases in vascular ROS levels were greater in Nrf2^–/–^ than in Nrf2^+/+^ mice (Ungvari et al., 2011a). Nrf2 deficient rats lead to the development of salt-induced oxidative stress, endothelial dysfunction, and microvessel rarefaction in normotensive rats (Priestley et al., 2016). A recent study conducted in individually perfused microvessels showed that Nrf2 deficient rats have higher plasma H_2_O_2_ levels than WT rats under both normal and STZ-induced diabetic conditions. Compared to WT rats, microvessels in Nrf2 deficient rats showed significantly higher basal permeability (measured by hydraulic conductivity, Lp) as well as increased permeability responses to PAF in both normal and STZ-induced diabetic rats (Xia et al., 2018). Importantly, the magnitude of the Lp increases mirrored the plasma H_2_O_2_ levels where higher ROS levels correlated with higher permeability increases when exposed to an inflammatory mediator, PAF. These studies indicate an important antioxidant role of Nrf2 under both normal and pathological conditions.

The important role of Nrf2 in the regulation of antioxidant responses led many studies to search pharmacological activators or inducers of Nrf2 to enhance Nrf2-driven antioxidant defense (Shih et al., 2005; Chen et al., 2015; Sharma et al., 2017). The induction of NRF2-driven antioxidant response in whole animal showed reduced ischemic damage from stroke and mitochondrial stress (Shih et al., 2005), and increased expression of Nrf2-driven genes protected the blood brain barrier after brain injury (Zhao et al., 2007). To date, increasing number of chemical compounds and plant natural compounds have been recognized to have antioxidant effect mediated by Nrf2-ARE related pathways from *in vivo* and *in vitro* studies (Chen et al., 2015). Some of the Nrf2 inducer supplements also showed promising anti-aging and anti-oxidant effects in clinical studies (Ghanim et al., 2011; Pergola et al., 2011). However, the conclusive evidence of their ability in improving clinical outcomes has been limited and warrants further investigation.

Current studies indicate that increased ROS and/or reduced antioxidant defense play important roles in priming endothelial cells and transitioning them into a phenotype with higher susceptibility to additional inflammatory stimuli or pathological conditions. Importantly, these studies open up a novel area of future investigations where direct upregulation of Nrf2 antioxidant defense system could prove to be a highly valuable therapeutic strategy against conditions associated with increased oxidative stress.

## Summary

**FIGURE 13:**
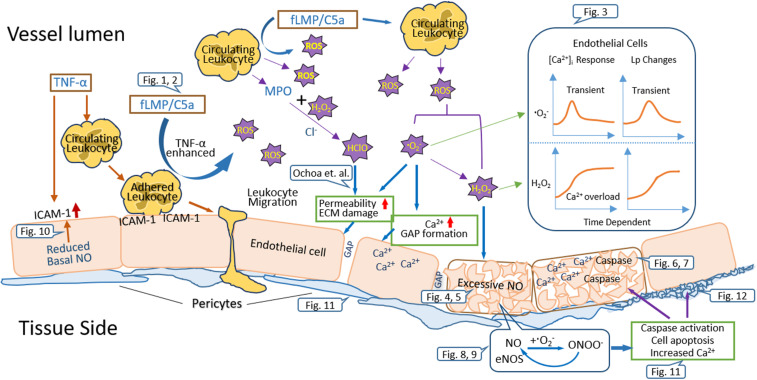
Schematic diagram summarizing the underlying mechanisms involved in leukocyte-dependent and ROS-mediated microvessel barrier dysfunction.

Clinical and experimental evidence indicate that increased vascular permeability contributes to many disease-associated vascular complications. It is thus important to identify the critical factors and underlying mechanisms involved in permeability increases. Increased production of ROS has been implicated in the pathogenesis of many cardiovascular diseases. As those outlined in this review, each species of ROS plays different roles in the regulation of vascular function. Importantly, one species of ROS can further amplify redox signaling, resulting in augmented oxidative and nitroxidative stress. Increasing number of studies recognize that H_2_O_2_, a relatively stable reactive oxygen metabolite, plays an important role in ROS-mediated vascular barrier dysfunction. For decades, increased ROS-induced vascular dysfunction has often been associated with reduced NO production through eNOS uncoupling. However, emerging evidence revealed that the H_2_O_2_-induced EC [Ca^2+^]_i_ overload, cell apoptosis, and progressively increased microvessel permeability were mediated by H_2_O_2_-induced excessive NO and NO-derived peroxynitrite. The interplay of ROS, NO, and RNS followed by a self-promoted amplification of nitroxidative stress contributes to ROS-mediated microvascular dysfunction, including early stage of pericyte loss in microvessels. The schematic diagram shown in Figure 13 summarizes the underlying mechanisms involved in leukocyte-dependent and ROS-mediated microvessel barrier dysfunction. Future studies on molecular mechanisms and gene regulations of ROS overproduction, targeted interference of the amplification cascade, or enhancement of the antioxidant would benefit the development of therapeutic strategies to alleviate ROS-mediated vascular barrier dysfunction, which underlies a variety of cardiovascular diseases.

## Author Contributions

All authors contributed to the writing, editing, and the effort is reflected by the author order and had final approval of the submitted version.

## Conflict of Interest

The authors declare that the research was conducted in the absence of any commercial or financial relationships that could be construed as a potential conflict of interest.

## References

[B1] Alexander J. S. Alexander B. C. Eppihimer L. A. Goodyear N. Haque R. Davis C. P. (2000). Inflammatory mediators induce sequestration of VE-cadherin in cultured human endothelial cells. *Inflammation* 24 99–113. 10.1023/a:1007025325451 10718113

[B2] Amin K. N. Bhakkiyalakshmi E. Ravichandran J. Sarada D. V. L. Mohanram R. K. (2019). The pivotal role of nuclear factor erythroid 2-related factor 2 in diabetes-induced endothelial dysfunction. *Pharmacol. Res.* 7:104601 10.1016/j.phrs.2019.104601 31838079

[B3] Anatoliotakis N. Deftereos S. Bouras G. Giannopoulos G. Tsounis D. Angelidis C. (2013). Myeloperoxidase: expressing inflammation and oxidative stress in cardiovascular disease. *Curr. Top. Med. Chem.* 13 115–138. 10.2174/1568026611313020004 23470074

[B4] Armulik A. Abramsson A. Betsholtz C. (2005). Endothelial/pericyte interactions. *Circ. Res.* 97 512–523. 16166562 10.1161/01.RES.0000182903.16652.d7

[B5] Armulik A. Genove G. Betsholtz C. (2011). Pericytes: developmental, physiological, and pathological perspectives, problems, and promises. *Dev. Cell* 21 193–215. 10.1016/j.devcel.2011.07.001 21839917

[B6] Armulik A. Genove G. Mae M. Nisancioglu M. H. Wallgard E. Niaudet C. (2010). Pericytes regulate the blood-brain barrier. *Nature* 468 557–561. 20944627 10.1038/nature09522

[B7] Atochin D. N. Huang P. L. (2010). Endothelial nitric oxide synthase transgenic models of endothelial dysfunction. *Pflugers. Arch.* 460 965–974. 10.1007/s00424-010-0867-4 20697735 PMC2975487

[B8] Az-Ma T. Fujii K. Yuge O. (1996). Self-limiting enhancement by nitric oxide of oxygen free radical-induced endothelial cell injury: evidence against the dual action of NO as hydroxyl radical donor/scavenger. *Br. J. Pharmacol.* 119 455–462. 10.1111/j.1476-5381.1996.tb15694.x 8894164 PMC1915714

[B9] Babior B. M. (1999). NADPH oxidase: an update. *Blood* 93 1464–1476. 10029572

[B10] Baldus S. Heeschen C. Meinertz T. Zeiher A. M. Eiserich J. P. Munzel T. (2003). Myeloperoxidase serum levels predict risk in patients with acute coronary syndromes. *Circulation* 108 1440–1445. 10.1161/01.CIR.0000090690.67322.51 12952835

[B11] Baluk P. Bertrand C. Geppetti P. Mcdonald D. M. Nadel J. A. (1995). NK1 receptors mediate leukocyte adhesion in neurogenic inflammation in the rat trachea. *Am. J. Physiol.* 268 L263–L269. 10.1152/ajplung.1995.268.2.L263 7864147

[B12] Baluk P. Bolton P. Hirata A. Thurston G. Mcdonald D. M. (1998). Endothelial gaps and adherent leukocytes in allergen-induced early- and late-phase plasma leakage in rat airways. *Am. J. Pathol.* 152 1463–1476. 9626051 PMC1858452

[B13] Baluk P. Hirata A. Thurston G. Fujiwara T. Neal C. R. Michel C. C. (1997). Endothelial gaps: time course of formation and closure in inflamed venules of rats. *Am. J. Physiol.* 272 L155–L170. 10.1152/ajplung.1997.272.1.L155 9038915

[B14] Barnard M. L. Matalon S. (1992). Mechanisms of extracellular reactive oxygen species injury to the pulmonary microvasculature. *J. Appl. Physiol.* 72 1724–1729. 10.1152/jappl.1992.72.5.1724 1601778

[B15] Barrett T. J. Hawkins C. L. (2012). Hypothiocyanous acid: benign or deadly? *Chem. Res. Toxicol.* 25 263–273. 10.1021/tx200219s 22053976

[B16] Beckman J. S. Beckman T. W. Chen J. Marshall P. A. Freeman B. A. (1990). Apparent hydroxyl radical production by peroxynitrite: implications for endothelial injury from nitric oxide and superoxide. *Proc. Natl. Acad. Sci. U.S.A.* 87 1620–1624. 10.1073/pnas.87.4.1620 2154753 PMC53527

[B17] Beckman J. S. Koppenol W. H. (1996). Nitric oxide, superoxide, and peroxynitrite: the good, the bad, and ugly. *Am. J. Physiol.* 271 C1424–C1437. 10.1152/ajpcell.1996.271.5.C1424 8944624

[B18] Bergers G. Song S. (2005). The role of pericytes in blood-vessel formation and maintenance. *Neuro. Oncol.* 7 452–464. 10.1215/S1152851705000232 16212810 PMC1871727

[B19] Berman R. S. Martin W. (1993). Arterial endothelial barrier dysfunction: actions of homocysteine and the hypoxanthine-xanthine oxidase free radical generating system. *Br. J. Pharmacol.* 108 920–926. 10.1111/j.1476-5381.1993.tb13487.x 8485631 PMC1908136

[B20] Bernard G. R. (1991). N-acetylcysteine in experimental and clinical acute lung injury. *Am. J. Med.* 91 54S–59S. 10.1016/0002-9343(91)90284-5 1928212

[B21] Bernard G. R. Wheeler A. P. Arons M. M. Morris P. E. Paz H. L. Russell J. A. (1997). A trial of antioxidants N-acetylcysteine and procysteine in ARDS. The Antioxidant in ARDS Study Group. *Chest* 112 164–172. 10.1378/chest.112.1.164 9228372

[B22] Bloomfield G. L. Holloway S. Ridings P. C. Fisher B. J. Blocher C. R. Sholley M. (1997). Pretreatment with inhaled nitric oxide inhibits neutrophil migration and oxidative activity resulting in attenuated sepsis-induced acute lung injury. *Crit. Care Med.* 25 584–593. 10.1097/00003246-199704000-00006 9142021

[B23] Bonavia A. Miller L. Kellum J. A. Singbartl K. (2017). Hemoadsorption corrects hyperresistinemia and restores anti-bacterial neutrophil function. *Intensive Care Med. Exp.* 5:36 10.1186/s40635-017-0150-5 PMC5544662 28779451

[B24] Boueiz A. Hassoun P. M. (2009). Regulation of endothelial barrier function by reactive oxygen and nitrogen species. *Microvasc. Res.* 77 26–34. 10.1016/j.mvr.2008.10.005 19041330

[B25] Brennan M. L. Penn M. S. Van Lente F. Nambi V. Shishehbor M. H. Aviles R. J. (2003). Prognostic value of myeloperoxidase in patients with chest pain. *N. Engl. J. Med.* 349 1595–1604. 14573731 10.1056/NEJMoa035003

[B26] Cai H. (2005a). Hydrogen peroxide regulation of endothelial function: origins, mechanisms, and consequences. *Cardiovasc. Res.* 68 26–36. 10.1016/j.cardiores.2005.06.021 16009356

[B27] Cai H. (2005b). NAD(P)H oxidase-dependent self-propagation of hydrogen peroxide and vascular disease. *Circ. Res.* 96 818–822. 10.1161/01.RES.0000163631.07205.fb 15860762

[B28] Cai H. Harrison D. G. (2000). Endothelial dysfunction in cardiovascular diseases: the role of oxidant stress. *Circ. Res.* 87 840–844. 10.1161/01.res.87.10.840 11073878

[B29] Cai H. Li Z. Davis M. E. Kanner W. Harrison D. G. (2003). Akt-dependent phosphorylation of serine 1179 and mitogen-activated protein kinase kinase/extracellular signal-regulated kinase 1/2 cooperatively mediate activation of the endothelial nitric-oxide synthase by hydrogen peroxide. *Mol. Pharmacol.* 63 325–331. 10.1124/mol.63.2.325 12527803

[B30] Carden D. L. Smith J. K. Korthuis R. J. (1990). Neutrophil-mediated microvascular dysfunction in postischemic canine skeletal muscle. Role of granulocyte adherence. *Circ. Res.* 66 1436–1444. 10.1161/01.res.66.5.1436 2159391

[B31] Chan J. Y. Kwong M. (2000). Impaired expression of glutathione synthetic enzyme genes in mice with targeted deletion of the Nrf2 basic-leucine zipper protein. *Biochim. Biophys. Acta* 1517 19–26. 10.1016/s0167-4781(00)00238-4 11118612

[B32] Chan K. Han X. D. Kan Y. W. (2001). An important function of Nrf2 in combating oxidative stress: detoxification of acetaminophen. *Proc. Natl. Acad. Sci. U.S.A.* 98 4611–4616. 10.1073/pnas.081082098 11287661 PMC31882

[B33] Chanas S. A. Jiang Q. Mcmahon M. Mcwalter G. K. Mclellan L. I. Elcombe C. R. (2002). Loss of the Nrf2 transcription factor causes a marked reduction in constitutive and inducible expression of the glutathione S-transferase Gsta1, Gsta2, Gstm1, Gstm2, Gstm3 and Gstm4 genes in the livers of male and female mice. *Biochem. J.* 365 405–416. 10.1042/BJ20020320 11991805 PMC1222698

[B34] Chen B. Lu Y. Chen Y. Cheng J. (2015). The role of Nrf2 in oxidative stress-induced endothelial injuries. *J. Endocrinol.* 225 R83–R99. 10.1530/JOE-14-0662 25918130

[B35] Clancy R. M. Leszczynska-Piziak J. Abramson S. B. (1992). Nitric oxide, an endothelial cell relaxation factor, inhibits neutrophil superoxide anion production via a direct action on the NADPH oxidase. *J. Clin. Invest.* 90 1116–1121. 10.1172/JCI115929 1325992 PMC329973

[B36] Cohen G. Ilic D. Raupachova J. Horl W. H. (2008). Resistin inhibits essential functions of polymorphonuclear leukocytes. *J. Immunol.* 181 3761–3768. 10.4049/jimmunol.181.6.3761 18768828

[B37] Condliffe A. M. Hawkins P. T. Stephens L. R. Haslett C. Chilvers E. R. (1998a). Priming of human neutrophil superoxide generation by tumour necrosis factor-alpha is signalled by enhanced phosphatidylinositol 3,4,5-trisphosphate but not inositol 1,4,5-trisphosphate accumulation. *FEBS Lett.* 439 147–151. 10.1016/s0014-5793(98)01358-1 9849896

[B38] Condliffe A. M. Kitchen E. Chilvers E. R. (1998b). Neutrophil priming: pathophysiological consequences and underlying mechanisms. *Clin. Sci.* 94 461–471. 10.1042/cs0940461 9682667

[B39] Cook N. L. Viola H. M. Sharov V. S. Hool L. C. Schoneich C. Davies M. J. (2012). Myeloperoxidase-derived oxidants inhibit sarco/endoplasmic reticulum Ca2+-ATPase activity and perturb Ca2+ homeostasis in human coronary artery endothelial cells. *Free Radic. Biol. Med.* 52 951–961. 10.1016/j.freeradbiomed.2011.12.001 22214747 PMC3736816

[B40] Dahlgren C. Karlsson A. (1999). Respiratory burst in human neutrophils. *J. Immunol. Methods* 232 3–14. 10618505 10.1016/s0022-1759(99)00146-5

[B41] Daiber A. (2010). Redox signaling (cross-talk) from and to mitochondria involves mitochondrial pores and reactive oxygen species. *Biochim. Biophys. Acta* 1797 897–906. 10.1016/j.bbabio.2010.01.032 20122895

[B42] Daiber A. Di Lisa F. Oelze M. Kroller-Schon S. Steven S. Schulz E. (2017). Crosstalk of mitochondria with NADPH oxidase via reactive oxygen and nitrogen species signalling and its role for vascular function. *Br. J. Pharmacol.* 174 1670–1689. 10.1111/bph.13403 26660451 PMC5446573

[B43] Daneman R. Zhou L. Kebede A. A. Barres B. A. (2010). Pericytes are required for blood-brain barrier integrity during embryogenesis. *Nature* 468 562–566. 10.1038/nature09513 20944625 PMC3241506

[B44] Daugherty A. Dunn J. L. Rateri D. L. Heinecke J. W. (1994). Myeloperoxidase, a catalyst for lipoprotein oxidation, is expressed in human atherosclerotic lesions. *J. Clin. Invest.* 94 437–444. 10.1172/JCI117342 8040285 PMC296328

[B45] Davies M. J. (2011). Myeloperoxidase-derived oxidation: mechanisms of biological damage and its prevention. *J. Clin. Biochem. Nutr.* 48 8–19. 10.3164/jcbn.11-006FR 21297906 PMC3022070

[B46] Davies M. J. Hawkins C. L. Pattison D. I. Rees M. D. (2008). Mammalian heme peroxidases: from molecular mechanisms to health implications. *Antioxid. Redox Signal.* 10 1199–1234. 10.1089/ars.2007.1927 18331199

[B47] Del Maestro R. F. Bjork J. Arfors K. E. (1981). Increase in microvascular permeability induced by enzymatically generated free radicals. I. In vivo study. *Microvasc. Res.* 22 239–254. 10.1016/0026-2862(81)90095-9 6895770

[B48] Del Maschio A. Zanetti A. Corada M. Rival Y. Ruco L. Lampugnani M. G. (1996). Polymorphonuclear leukocyte adhesion triggers the disorganization of endothelial cell-to-cell adherens junctions. *J. Cell Biol.* 135 497–510. 10.1083/jcb.135.2.497 8896605 PMC2121047

[B49] Dente C. J. Steffes C. P. Speyer C. Tyburski J. G. (2001). Pericytes augment the capillary barrier in in vitro cocultures. *J. Surg. Res.* 97 85–91. 10.1006/jsre.2001.6117 11319886

[B50] Djordjevic T. Pogrebniak A. Belaiba R. S. Bonello S. Wotzlaw C. Acker H. (2005). The expression of the NADPH oxidase subunit p22phox is regulated by a redox-sensitive pathway in endothelial cells. *Free Radic. Biol. Med.* 38 616–630. 10.1016/j.freeradbiomed.2004.09.036 15683718

[B51] Doan T. N. Gentry D. L. Taylor A. A. Elliott S. J. (1994). Hydrogen peroxide activates agonist-sensitive Ca(2+)-flux pathways in canine venous endothelial cells. *Biochem. J.* 297(Pt 1), 209–215. 10.1042/bj2970209 8280101 PMC1137812

[B52] Dreher D. Junod A. F. (1995). Differential effects of superoxide, hydrogen peroxide, and hydroxyl radical on intracellular calcium in human endothelial cells. *J. Cell Physiol.* 162 147–153. 10.1002/jcp.1041620118 7814447

[B53] Droge W. (2002). Free radicals in the physiological control of cell function. *Physiol. Rev.* 82 47–95. 10.1152/physrev.00018.2001 11773609

[B54] Edelman D. A. Jiang Y. Tyburski J. Wilson R. F. Steffes C. (2006). Pericytes and their role in microvasculature homeostasis. *J. Surg. Res.* 135 305–311. 16930620 10.1016/j.jss.2006.06.010

[B55] Eiserich J. P. Baldus S. Brennan M. L. Ma W. Zhang C. Tousson A. (2002). Myeloperoxidase, a leukocyte-derived vascular NO oxidase. *Science* 296 2391–2394. 10.1126/science.1106830 12089442

[B56] El-Benna J. Hurtado-Nedelec M. Marzaioli V. Marie J. C. Gougerot-Pocidalo M. A. Dang P. M. (2016). Priming of the neutrophil respiratory burst: role in host defense and inflammation. *Immunol. Rev.* 273 180–193. 10.1111/imr.12447 27558335

[B57] Eschwege P. Paradis V. Conti M. Holstege A. Richet F. Deteve J. (1999). In situ detection of lipid peroxidation by-products as markers of renal ischemia injuries in rat kidneys. *J. Urol.* 162 553–557. 10411087

[B58] Ferland-McCollough D. Slater S. Richard J. Reni C. Mangialardi G. (2017). Pericytes, an overlooked player in vascular pathobiology. *Pharmacol. Ther.* 171 30–42. 10.1016/j.pharmthera.2016.11.008 27916653 PMC6008604

[B59] Fernandez-Hernando C. Yu J. Suarez Y. Rahner C. Davalos A. Lasuncion M. A. (2009). Genetic evidence supporting a critical role of endothelial caveolin-1 during the progression of atherosclerosis. *Cell Metab.* 10 48–54. 10.1016/j.cmet.2009.06.003 19583953 PMC2735117

[B60] Finkel T. (1998). Oxygen radicals and signaling. *Curr. Opin. Cell Biol.* 10 248–253. 9561849 10.1016/s0955-0674(98)80147-6

[B61] Forstermann U. (2008). Oxidative stress in vascular disease: causes, defense mechanisms and potential therapies. *Nat. Clin. Pract. Cardiovasc. Med.* 5 338–349. 10.1038/ncpcardio1211 18461048

[B62] Forstermann U. Xia N. Li H. (2017). Roles of vascular oxidative stress and nitric oxide in the pathogenesis of atherosclerosis. *Circ. Res.* 120 713–735. 10.1161/CIRCRESAHA.116.309326 28209797

[B63] Fritzsche C. Schleicher U. Bogdan C. (2010). Endothelial nitric oxide synthase limits the inflammatory response in mouse cutaneous leishmaniasis. *Immunobiology* 215 826–832. 10.1016/j.imbio.2010.05.022 20576313

[B64] Furchgott R. F. Vanhoutte P. M. (1989). Endothelium-derived relaxing and contracting factors. *FASEB J.* 3 2007–2018. 10.1161/01.hyp.19.5.442 2545495

[B65] Gaengel K. Genove G. Armulik A. Betsholtz C. (2009). Endothelial-mural cell signaling in vascular development and angiogenesis. *Arterioscler. Thromb. Vasc. Biol.* 29 630–638. 10.1161/ATVBAHA.107.161521 19164813

[B66] Gao F. Lucke-Wold B. P. Li X. Logsdon A. F. Xu L. C. Xu S. (2017). Reduction of endothelial nitric oxide increases the adhesiveness of constitutive endothelial membrane ICAM-1 through src-mediated phosphorylation. *Front. Physiol.* 8:1124 10.3389/fphys.2017.01124 PMC5768177 29367846

[B67] Gerhardt H. Betsholtz C. (2003). Endothelial-pericyte interactions in angiogenesis. *Cell Tissue Res.* 314 15–23. 12883993 10.1007/s00441-003-0745-x

[B68] Ghanim H. Sia C. L. Korzeniewski K. Lohano T. Abuaysheh S. Marumganti A. (2011). A resveratrol and polyphenol preparation suppresses oxidative and inflammatory stress response to a high-fat, high-carbohydrate meal. *J. Clin. Endocrinol. Metab.* 96 1409–1414. 10.1210/jc.2010-1812 21289251 PMC3085195

[B69] Granger D. N. Kvietys P. R. (2015). Reperfusion injury and reactive oxygen species: the evolution of a concept. *Redox Biol.* 6 524–551. 10.1016/j.redox.2015.08.020 26484802 PMC4625011

[B70] Gupta M. P. Ober M. D. Patterson C. Al-Hassani M. Natarajan V. Hart C. M. (2001). Nitric oxide attenuates H(2)O(2)-induced endothelial barrier dysfunction: mechanisms of protection. *Am. J. Physiol. Lung Cell. Mol. Physiol.* 280 L116–L126. 10.1152/ajplung.2001.280.1.L116 11133501

[B71] Gupta M. P. Steinberg H. O. Hart C. M. (1998). H2O2 causes endothelial barrier dysfunction without disrupting the arginine-nitric oxide pathway. *Am. J. Physiol.* 274 L508–L516. 10.1152/ajplung.1998.274.4.L508 9575868

[B72] Hampton M. B. Kettle A. J. Winterbourn C. C. (1998). Inside the neutrophil phagosome: oxidants, myeloperoxidase, and bacterial killing. *Blood* 92 3007–3017. 9787133

[B73] Harris N. R. Benoit J. N. Granger D. N. (1993). Capillary filtration during acute inflammation: role of adherent neutrophils. *Am. J. Physiol.* 265 H1623–H1628. 10.1152/ajpheart.1993.265.5.H1623 7902014

[B74] Harrison D. Griendling K. K. Landmesser U. Hornig B. Drexler H. (2003). Role of oxidative stress in atherosclerosis. *Am. J. Cardiol.* 91 7A–11A. 10.1016/s0002-9149(02)03144-2 12645638

[B75] Hartmann D. A. Underly R. G. Grant R. I. Watson A. N. Lindner V. Shih A. Y. (2015). Pericyte structure and distribution in the cerebral cortex revealed by high-resolution imaging of transgenic mice. *Neurophotonics* 2:41402 10.1117/1.NPh.2.4.041402 PMC4478963 26158016

[B76] Hatakeyama T. Pappas P. J. Hobson R. W. II Boric M. P. Sessa W. C. Duran W. N. (2006). Endothelial nitric oxide synthase regulates microvascular hyperpermeability in vivo. *J. Physiol.* 574 275–281. 10.1113/jphysiol.2006.108175 16675496 PMC1817804

[B77] He P. (2010). Leucocyte/endothelium interactions and microvessel permeability: coupled or uncoupled? *Cardiovasc. Res.* 87 281–290. 10.1093/cvr/cvq140 20472564 PMC2895544

[B78] He P. Liu B. Curry F. E. (1997). Effect of nitric oxide synthase inhibitors on endothelial [Ca2+]i and microvessel permeability. *Am. J. Physiol.* 272 H176–H185. 10.1152/ajpheart.1997.272.1.H176 9038936

[B79] He P. Zhang H. Zhu L. Jiang Y. Zhou X. (2006). Leukocyte-platelet aggregate adhesion and vascular permeability in intact microvessels: role of activated endothelial cells. *Am. J. Physiol. Heart Circ. Physiol.* 291 H591–H599. 10.1152/ajpheart.01228.2005 16517944

[B80] Hecquet C. M. Ahmmed G. U. Vogel S. M. Malik A. B. (2008). Role of TRPM2 channel in mediating H2O2-induced Ca2+ entry and endothelial hyperpermeability. *Circ. Res.* 102 347–355. 10.1161/CIRCRESAHA.107.160176 18048770

[B81] Henricks P. A. Nijkamp F. P. (2001). Reactive oxygen species as mediators in asthma. *Pulm. Pharmacol. Ther.* 14 409–420. 10.1006/pupt.2001.0319 11782121

[B82] Hill J. Rom S. Ramirez S. H. Persidsky Y. (2014). Emerging roles of pericytes in the regulation of the neurovascular unit in health and disease. *J. Neuroimmun. Pharmacol.* 9 591–605. 10.1007/s11481-014-9557-x PMC4209199 25119834

[B83] Hurley J. V. (1964). Acute inflammation: the effect of concurrent leucocytic emigration and increased permeability on particle retention by the vascular wall. *Br. J. Exp. Pathol.* 45 627–633. 14245163 PMC2093666

[B84] Ishikawa M. Cooper D. Arumugam T. V. Zhang J. H. Nanda A. Granger D. N. (2004). Platelet-leukocyte-endothelial cell interactions after middle cerebral artery occlusion and reperfusion. *J. Cereb. Blood Flow Metab.* 24 907–915. 10.1097/01.WCB.0000132690.96836.7F 15362721

[B85] Jin B. Y. Lin A. J. Golan D. E. Michel T. (2012). MARCKS protein mediates hydrogen peroxide regulation of endothelial permeability. *Proc. Natl. Acad. Sci. U.S.A.* 109 14864–14869. 10.1073/pnas.1204974109 22927426 PMC3443126

[B86] Johnson A. Phillips P. Hocking D. Tsan M. F. Ferro T. (1989). Protein kinase inhibitor prevents pulmonary edema in response to H2O2. *Am. J. Physiol.* 256 H1012–H1022. 10.1152/ajpheart.1989.256.4.H1012 2705544

[B87] Kadambi A. Skalak T. C. (2000). Role of leukocytes and tissue-derived oxidants in short-term skeletal muscle ischemia-reperfusion injury. *Am. J. Physiol. Heart Circ. Physiol.* 278 H435–H443. 10.1152/ajpheart.2000.278.2.H435 10666073

[B88] Kalogeris T. Baines C. P. Krenz M. Korthuis R. J. (2016). Ischemia/Reperfusion. *Compr. Physiol.* 7 113–170. 28135002 10.1002/cphy.c160006 PMC5648017

[B89] Kamata H. Hirata H. (1999). Redox regulation of cellular signalling. *Cell Signal.* 11 1–14. 10206339 10.1016/s0898-6568(98)00037-0

[B90] Kavanagh B. P. Mouchawar A. Goldsmith J. Pearl R. G. (1994). Effects of inhaled NO and inhibition of endogenous NO synthesis in oxidant-induced acute lung injury. *J. Appl. Physiol.* 76 1324–1329. 10.1152/jappl.1994.76.3.1324 8005878

[B91] Kelley C. D’amore P. Hechtman H. B. Shepro D. (1988). Vasoactive hormones and cAMP affect pericyte contraction and stress fibres in vitro. *J. Muscle Res. Cell Motil.* 9 184–194. 10.1007/BF01773740 2458383

[B92] Kennedy T. P. Rao N. V. Hopkins C. Pennington L. Tolley E. Hoidal J. R. (1989). Role of reactive oxygen species in reperfusion injury of the rabbit lung. *J. Clin. Invest.* 83 1326–1335. 10.1172/JCI114019 2467923 PMC303825

[B93] Klebanoff S. J. (2005). Myeloperoxidase: friend and foe. *J. Leukoc. Biol.* 77 598–625. 15689384 10.1189/jlb.1204697

[B94] Knepler J. L. Jr. Taher L. N. Gupta M. P. Patterson C. Pavalko F. (2001). Peroxynitrite causes endothelial cell monolayer barrier dysfunction. *Am. J. Physiol. Cell Physiol.* 281 C1064–C1075. 10.1152/ajpcell.2001.281.3.C1064 11502585

[B95] Korthuis R. J. Grisham M. B. Granger D. N. (1988). Leukocyte depletion attenuates vascular injury in postischemic skeletal muscle. *Am. J. Physiol.* 254 H823–H827. 10.1152/ajpheart.1988.254.5.H823 3364586

[B96] Kroller-Schon S. Steven S. Kossmann S. Scholz A. Daub S. Oelze M. (2014). Molecular mechanisms of the crosstalk between mitochondria and NADPH oxidase through reactive oxygen species-studies in white blood cells and in animal models. *Antioxid. Redox Signal.* 20 247–266. 23845067 10.1089/ars.2012.4953 PMC3887465

[B97] Kubes P. Granger D. N. (1992). Nitric oxide modulates microvascular permeability. *Am. J. Physiol.* 262 H611–H615. 10.1152/ajpheart.1996.271.4.H1702 1539722

[B98] Kubes P. Kanwar S. Niu X. F. Gaboury J. P. (1993). Nitric oxide synthesis inhibition induces leukocyte adhesion via superoxide and mast cells. *FASEB J.* 7 1293–1299. 10.1096/fasebj.7.13.8405815 8405815

[B99] Kubes P. Suzuki M. Granger D. N. (1990a). Modulation of PAF-induced leukocyte adherence and increased microvascular permeability. *Am. J. Physiol.* 259 G859–G864. 10.1152/ajpgi.1990.259.5.G859 2240225

[B100] Kubes P. Suzuki M. Granger D. N. (1990b). Platelet-activating factor-induced microvascular dysfunction: role of adherent leukocytes. *Am. J. Physiol.* 258 G158–G163. 10.1152/ajpgi.1990.258.1.G158 2301577

[B101] Kubes P. Suzuki M. Granger D. N. (1991). Nitric oxide: an endogenous modulator of leukocyte adhesion. *Proc. Natl. Acad. Sci. U.S.A.* 88 4651–4655. 10.1073/pnas.88.11.4651 1675786 PMC51723

[B102] Kurose I. Wolf R. Grisham M. B. Aw T. Y. Specian R. D. Granger D. N. (1995a). Microvascular responses to inhibition of nitric oxide production. Role of active oxidants. *Circ. Res.* 76 30–39. 10.1161/01.res.76.1.30 7528112

[B103] Kurose I. Wolf R. Grisham M. B. Granger D. N. (1995b). Effects of an endogenous inhibitor of nitric oxide synthesis on postcapillary venules. *Am. J. Physiol.* 268 H2224–H2231. 10.1152/ajpheart.1995.268.6.H2224 7541959

[B104] Kvietys P. R. Granger D. N. (2012). Role of reactive oxygen and nitrogen species in the vascular responses to inflammation. *Free Radic. Biol. Med.* 52 556–592. 10.1016/j.freeradbiomed.2011.11.002 22154653 PMC3348846

[B105] La Rocca G. Di Stefano A. Eleuteri E. Anzalone R. Magno F. Corrao S. (2009). Oxidative stress induces myeloperoxidase expression in endocardial endothelial cells from patients with chronic heart failure. *Basic Res. Cardiol.* 104 307–320. 10.1007/s00395-008-0761-9 19030913

[B106] Lacy F. Kailasam M. T. O’connor D. T. Schmid-Schonbein G. W. Parmer R. J. (2000). Plasma hydrogen peroxide production in human essential hypertension: role of heredity, gender, and ethnicity. *Hypertension* 36 878–884. 10.1161/01.hyp.36.5.878 11082160

[B107] Landmesser U. Dikalov S. Price S. R. Mccann L. Fukai T. Holland S. M. (2003). Oxidation of tetrahydrobiopterin leads to uncoupling of endothelial cell nitric oxide synthase in hypertension. *J. Clin. Invest.* 111 1201–1209. 10.1172/JCI14172 12697739 PMC152929

[B108] Landmesser U. Spiekermann S. Preuss C. Sorrentino S. Fischer D. Manes C. (2007). Angiotensin II induces endothelial xanthine oxidase activation: role for endothelial dysfunction in patients with coronary disease. *Arterioscler. Thromb. Vasc. Biol.* 27 943–948. 10.1161/01.ATV.0000258415.32883.bf 17234726

[B109] Lau D. Baldus S. (2006). Myeloperoxidase and its contributory role in inflammatory vascular disease. *Pharmacol. Ther.* 111 16–26. 10.1016/j.pharmthera.2005.06.023 16476484

[B110] Lee K. S. Kim S. R. Park S. J. Park H. S. Min K. H. Lee M. H. (2006). Hydrogen peroxide induces vascular permeability via regulation of vascular endothelial growth factor. *Am. J. Respir. Cell Mol. Biol.* 35 190–197. 10.1165/rcmb.2005-0482OC 16574943

[B111] Leff J. A. Wilke C. P. Hybertson B. M. Shanley P. F. Beehler C. J. Repine J. E. (1993). Postinsult treatment with N-acetyl-L-cysteine decreases IL-1-induced neutrophil influx and lung leak in rats. *Am. J. Physiol.* 265 L501–L506. 10.1152/ajplung.1993.265.5.L501 8238538

[B112] Li H. Forstermann U. (2013). Uncoupling of endothelial NO synthase in atherosclerosis and vascular disease. *Curr. Opin. Pharmacol.* 13 161–167. 10.1016/j.coph.2013.01.006 23395155

[B113] Li H. Horke S. Forstermann U. (2014). Vascular oxidative stress, nitric oxide and atherosclerosis. *Atherosclerosis* 237 208–219. 25244505 10.1016/j.atherosclerosis.2014.09.001

[B114] Li W. G. Miller F. J. Jr. Zhang H. J. Spitz D. R. Oberley L. W. (2001). H(2)O(2)-induced O(2) production by a non-phagocytic NAD(P)H oxidase causes oxidant injury. *J. Biol. Chem.* 276 29251–29256. 10.1074/jbc.M102124200 11358965 PMC3974124

[B115] Lum H. (2001). Lysophospholipids in the regulation of endothelial barrier function. *Am. J. Physiol. Lung Cell. Mol. Physiol.* 281 L1335–L1336. 10.1152/ajplung.2001.281.6.L1335 11704527

[B116] Lum H. Roebuck K. A. (2001). Oxidant stress and endothelial cell dysfunction. *Am. J. Physiol. Cell. Physiol.* 280 C719–C741. 10.1152/ajpcell.2001.280.4.C719 11245588

[B117] Lush C. W. Kvietys P. R. (2000). Microvascular dysfunction in sepsis. *Microcirculation* 7 83–101. 10802851 10.1038/sj.mn.7300096

[B118] Ma Q. (2013). Role of nrf2 in oxidative stress and toxicity. *Annu. Rev. Pharmacol. Toxicol.* 53 401–426. 10.1002/iub.2066 23294312 PMC4680839

[B119] Madamanchi N. R. Vendrov A. Runge M. S. (2005). Oxidative stress and vascular disease. *Arterioscler. Thromb. Vasc. Biol.* 25 29–38. 15539615 10.1161/01.ATV.0000150649.39934.13

[B120] Mayhan W. G. (1994). Nitric oxide accounts for histamine-induced increases in macromolecular extravasation. *Am. J. Physiol.* 266 H2369–H2373. 10.1152/ajpheart.1994.266.6.H2369 7912900

[B121] Mcdonald D. M. (1994). Endothelial gaps and permeability of venules in rat tracheas exposed to inflammatory stimuli. *Am. J. Physiol.* 266 L61–L83. 10.1152/ajplung.1994.266.1.L61 7508201

[B122] Mcelroy M. C. Wiener-Kronish J. P. Miyazaki H. Sawa T. Modelska K. Dobbs L. G. (1997). Nitric oxide attenuates lung endothelial injury caused by sublethal hyperoxia in rats. *Am. J. Physiol.* 272 L631–L638. 10.1152/ajplung.1997.272.4.L631 9142935

[B123] Mcleish K. R. Merchant M. L. Creed T. M. Tandon S. Barati M. T. Uriarte S. M. (2017). Frontline science: tumor necrosis factor-alpha stimulation and priming of human neutrophil granule exocytosis. *J. Leukoc. Biol.* 102 19–29. 10.1189/jlb.3HI0716-293RR 28096297 PMC5470837

[B124] Mcnally J. S. Davis M. E. Giddens D. P. Saha A. Hwang J. Dikalov S. (2003). Role of xanthine oxidoreductase and NAD(P)H oxidase in endothelial superoxide production in response to oscillatory shear stress. *Am. J. Physiol. Heart Circ. Physiol.* 285 H2290–H2297. 10.1152/ajpheart.00515.2003 12958034

[B125] Mcquaid K. E. Keenan A. K. (1997). Endothelial barrier dysfunction and oxidative stress: roles for nitric oxide? *Exp. Physiol.* 82 369–376. 10.1113/expphysiol.1997.sp004032 9129951

[B126] Mcquaid K. E. Smyth E. M. Keenan A. K. (1996). Evidence for modulation of hydrogen peroxide-induced endothelial barrier dysfunction by nitric oxide in vitro. *Eur. J. Pharmacol.* 307 233–241. 10.1016/0014-2999(96)00271-3 8832226

[B127] Mcsweeney S. R. Warabi E. Siow R. C. (2016). Nrf2 as an endothelial mechanosensitive transcription factor: going with the flow. *Hypertension* 67 20–29. 10.1161/HYPERTENSIONAHA.115.06146 26597822

[B128] Meuwese M. C. Stroes E. S. Hazen S. L. Van Miert J. N. Kuivenhoven J. A. Schaub R. G. (2007). Serum myeloperoxidase levels are associated with the future risk of coronary artery disease in apparently healthy individuals: the EPIC-Norfolk prospective population study. *J. Am. Coll. Cardiol.* 50 159–165. 10.1016/j.jacc.2007.03.033 17616301

[B129] Miller L. Singbartl K. Chroneos Z. C. Ruiz-Velasco V. Lang C. H. Bonavia A. (2019). Resistin directly inhibits bacterial killing in neutrophils. *Intensive Care Med. Exp.* 7:30 10.1186/s40635-019-0257-y PMC6542889 31147868

[B130] Miralda I. Uriarte S. M. Mcleish K. R. (2017). Multiple phenotypic changes define neutrophil priming. *Front. Cell. Infect. Microbiol.* 7:217 10.3389/fphys.2017.0217 PMC5447094 28611952

[B131] Mocatta T. J. Pilbrow A. P. Cameron V. A. Senthilmohan R. Frampton C. M. Richards A. M. (2007). Plasma concentrations of myeloperoxidase predict mortality after myocardial infarction. *J. Am. Coll. Cardiol.* 49 1993–2000. 10.1016/j.jacc.2007.02.040 17512353

[B132] Modig J. Sandin R. (1988). Haematological, physiological and survival data in a porcine model of adult respiratory distress syndrome induced by endotoxaemia. Effects of treatment with N-acetylcysteine. *Acta Chir. Scand.* 154 169–177. 3287812

[B133] Moll T. Dejana E. Vestweber D. (1998). In vitro degradation of endothelial catenins by a neutrophil protease. *J. Cell Biol.* 140 403–407. 10.1083/jcb.140.2.403 9442115 PMC2132583

[B134] Moncada S. Higgs E. A. (2006). The discovery of nitric oxide and its role in vascular biology. *Br. J. Pharmacol.* 147(Suppl. 1), S193–S201. 10.1038/sj.bjp.0706458 16402104 PMC1760731

[B135] Moncada S. Palmer R. M. Higgs E. A. (1991). Nitric oxide: physiology, pathophysiology, and pharmacology. *Pharmacol. Rev.* 43 109–142. 1852778

[B136] Montezano A. C. Touyz R. M. (2012). Reactive oxygen species and endothelial function–role of nitric oxide synthase uncoupling and Nox family nicotinamide adenine dinucleotide phosphate oxidases. *Basic Clin. Pharmacol. Toxicol.* 110 87–94. 10.1111/j.1742-7843.2011.00785.x 21883939

[B137] Motohashi H. Yamamoto M. (2004). Nrf2-Keap1 defines a physiologically important stress response mechanism. *Trends Mol. Med.* 10 549–557. 10.1016/j.molmed.2004.09.003 15519281

[B138] Mueller C. F. Laude K. Mcnally J. S. Harrison D. G. (2005). ATVB in focus: redox mechanisms in blood vessels. *Arterioscler. Thromb. Vasc. Biol.* 25 274–278. 10.1161/01.ATV.0000153515.72375.3b 15514203

[B139] Mundi S. Massaro M. Scoditti E. Carluccio M. A. Van Hinsbergh V. W. M. Iruela-Arispe M. L. (2017). Endothelial permeability, Ldl deposition, and cardiovascular risk factors - a review. *Cardiovasc. Res.* 114 35–52. 10.1093/cvr/cvx226 PMC7729208 29228169

[B140] Murfee W. L. Skalak T. C. Peirce S. M. (2005). Differential arterial/venous expression of NG2 proteoglycan in perivascular cells along microvessels: identifying a venule-specific phenotype. *Microcirculation* 12 151–160. 10.1080/10739680590904955 15824037

[B141] Ndrepepa G. (2019). Myeloperoxidase - A bridge linking inflammation and oxidative stress with cardiovascular disease. *Clin. Chim. Acta* 493 36–51. 10.1016/j.cca.2019.02.022 30797769

[B142] Neumann P. Gertzberg N. Vaughan E. Weisbrot J. Woodburn R. Lambert W. (2006). Peroxynitrite mediates TNF-alpha-induced endothelial barrier dysfunction and nitration of actin. *Am. J. Physiol. Lung Cell. Mol. Physiol.* 290 L674–L684. 10.1152/ajplung.00391.2005 16284212

[B143] Nguyen T. Nioi P. Pickett C. B. (2009). The Nrf2-antioxidant response element signaling pathway and its activation by oxidative stress. *J. Biol. Chem.* 284 13291–13295. 10.1074/jbc.R900010200 19182219 PMC2679427

[B144] Nicholls S. J. Hazen S. L. (2009). Myeloperoxidase, modified lipoproteins, and atherogenesis. *J. Lipid Res.* 50(Suppl.), S346–S351. 10.1194/jlr.R800086-JLR200 19091698 PMC2674690

[B145] Niu X. F. Smith C. W. Kubes P. (1994). Intracellular oxidative stress induced by nitric oxide synthesis inhibition increases endothelial cell adhesion to neutrophils. *Circ. Res.* 74 1133–1140. 10.1161/01.res.74.6.1133 7910528

[B146] Ochoa L. Waypa G. Mahoney J. R. Jr. Rodriguez L. Minnear F. L. (1997). Contrasting effects of hypochlorous acid and hydrogen peroxide on endothelial permeability: prevention with cAMP drugs. *Am. J. Respir. Crit. Care Med.* 156 1247–1255. 10.1164/ajrccm.156.4.96-10115 9351629

[B147] Okayama N. Grisham M. B. Kevil C. G. Eppihimer L. A. Wink D. A. Alexander J. S. (1999). Effect of reactive oxygen metabolites on endothelial permeability: role of nitric oxide and iron. *Microcirculation* 6 107–116. 10466113

[B148] Okayama N. Kevil C. G. Correia L. Jourd’heuil D. Itoh M. Grisham M. B. (1997). Nitric oxide enhances hydrogen peroxide-mediated endothelial permeability in vitro. *Am. J. Physiol.* 273 C1581–C1587. 10.1152/ajpcell.1997.273.5.C1581 9374643

[B149] Pacher P. Beckman J. S. Liaudet L. (2007). Nitric oxide and peroxynitrite in health and disease. *Physiol. Rev.* 87 315–424. 10.1152/physrev.00029.2006 17237348 PMC2248324

[B150] Parks D. A. Shah A. K. Granger D. N. (1984). Oxygen radicals: effects on intestinal vascular permeability. *Am. J. Physiol.* 247 G167–G170. 10.1152/ajpgi.1984.247.2.G167 6087676

[B151] Parsons P. E. Worthen G. S. Moore E. E. Tate R. M. Henson P. M. (1989). The association of circulating endotoxin with the development of the adult respiratory distress syndrome. *Am. Rev. Respir. Dis.* 140, 294–301. 10.1164/ajrccm/140.2.294 2764364

[B152] Pergola P. E. Raskin P. Toto R. D. Meyer C. J. Huff J. W. Grossman E. B. (2011). Bardoxolone methyl and kidney function in CKD with type 2 diabetes. *N. Engl. J. Med.* 365 327–336. 10.1056/NEJMoa1105351 21699484

[B153] Pinsky M. R. Vincent J.-L. Deviere J. Alegre M. Kahn R. J. Dupont E. (1993). Serum cytokine levels in human septic shock. Relation to multiple-system organ failure and mortality. *Chest* 103, 565–575. 10.1378/chest.103.2.565 8432155

[B154] Ponnuswamy P. Schrottle A. Ostermeier E. Gruner S. Huang P. L. Ertl G. (2012). eNOS protects from atherosclerosis despite relevant superoxide production by the enzyme in apoE mice. *PLoS One* 7:e30193 10.1371/journal.pone.0030193 PMC3264598 22291917

[B155] Poss W. B. Timmons O. D. Farrukh I. S. Hoidal J. R. Michael J. R. (1995). Inhaled nitric oxide prevents the increase in pulmonary vascular permeability caused by hydrogen peroxide. *J. Appl. Physiol.* 79 886–891. 10.1152/jappl.1995.79.3.886 8567532

[B156] Potenza M. A. Gagliardi S. Nacci C. Carratu M. R. Montagnani M. (2009). Endothelial dysfunction in diabetes: from mechanisms to therapeutic targets. *Curr. Med. Chem.* 16 94–112. 10.2174/092986709787002853 19149564

[B157] Potera R. M. Jensen M. J. Hilkin B. M. South G. K. Hook J. S. Gross E. A. (2016). Neutrophil azurophilic granule exocytosis is primed by TNF-alpha and partially regulated by NADPH oxidase. *Innate Immun.* 22 635–646. 10.1177/1753425916668980 27655046 PMC6805152

[B158] Priestley J. R. Kautenburg K. E. Casati M. C. Endres B. T. Geurts A. M. Lombard J. H. (2016). The NRF2 knockout rat: a new animal model to study endothelial dysfunction, oxidant stress, and microvascular rarefaction. *Am. J. Physiol. Heart Circ. Physiol.* 310 H478–H487. 10.1152/ajpheart.00586.2015 26637559 PMC4796617

[B159] Radi R. (2013). Protein tyrosine nitration: biochemical mechanisms and structural basis of functional effects. *Acc. Chem. Res.* 46 550–559. 10.1021/ar300234c 23157446 PMC3577981

[B160] Radi R. Beckman J. S. Bush K. M. Freeman B. A. (1991). Peroxynitrite oxidation of sulfhydryls. The cytotoxic potential of superoxide and nitric oxide. *J. Biol. Chem.* 266 4244–4250. 1847917

[B161] Radomski M. W. Palmer R. M. Moncada S. (1990a). An L-arginine/nitric oxide pathway present in human platelets regulates aggregation. *Proc. Natl. Acad. Sci. U.S.A.* 87 5193–5197. 10.1073/pnas.87.13.5193 1695013 PMC54288

[B162] Radomski M. W. Palmer R. M. Moncada S. (1990b). Characterization of the L-arginine:nitric oxide pathway in human platelets. *Br. J. Pharmacol.* 101 325–328. 10.1111/j.1476-5381.1990.tb12709.x 1701676 PMC1917694

[B163] Rhee S. G. (1999). Redox signaling: hydrogen peroxide as intracellular messenger. *Exp. Mol. Med.* 31 53–59. 10.1038/emm.1999.9 10410302

[B164] Riobo N. A. Clementi E. Melani M. Boveris A. Cadenas E. Moncada S. (2001). Nitric oxide inhibits mitochondrial NADH:ubiquinone reductase activity through peroxynitrite formation. *Biochem. J.* 359 139–145. 10.1042/0264-6021:3590139 11563977 PMC1222129

[B165] Rucker H. K. Wynder H. J. Thomas W. E. (2000). Cellular mechanisms of CNS pericytes. *Brain Res. Bull.* 51 363–369. 10715555 10.1016/s0361-9230(99)00260-9

[B166] Sagare A. P. Bell R. D. Zhao Z. Ma Q. Winkler E. A. Ramanathan A. (2013). Pericyte loss influences Alzheimer-like neurodegeneration in mice. *Nat. Commun.* 4:2932 10.1038/ncomms3932 PMC3945879 24336108

[B167] Salvemini D. Wang Z. Q. Stern M. K. Currie M. G. Misko T. P. (1998). Peroxynitrite decomposition catalysts: therapeutics for peroxynitrite-mediated pathology. *Proc. Natl. Acad. Sci. U.S.A.* 95 2659–2663. 10.1073/pnas.95.5.2659 9482943 PMC19452

[B168] Sanchez F. A. Rana R. Kim D. D. Iwahashi T. Zheng R. F. Lal B. K. (2009). Internalization of eNOS and NO delivery to subcellular targets determine agonist-induced hyperpermeability. *Proc. Natl. Acad. Sci. U.S.A.* 106 6849–6853. 10.1073/pnas.0812694106 19342481 PMC2672508

[B169] Sasaki M. Bharwani S. Jordan P. Elrod J. W. Grisham M. B. Jackson T. H. (2003). Increased disease activity in eNOS-deficient mice in experimental colitis. *Free Radic. Biol. Med.* 35 1679–1687. 10.1016/j.freeradbiomed.2003.09.016 14680690

[B170] Schindhelm R. K. Van Der Zwan L. P. Teerlink T. Scheffer P. G. (2009). Myeloperoxidase: a useful biomarker for cardiovascular disease risk stratification? *Clin. Chem.* 55 1462–1470. 10.1373/clinchem.2009.126029 19556446

[B171] Schultz J. Kaminker K. (1962). Myeloperoxidase of the leucocyte of normal human blood. I. Content and localization. *Arch. Biochem. Biophys.* 96 465–467. 10.1016/0003-9861(62)90321-1 13909511

[B172] Seeger W. Hansen T. Rossig R. Schmehl T. Schutte H. Kramer H. J. (1995). Hydrogen peroxide-induced increase in lung endothelial and epithelial permeability–effect of adenylate cyclase stimulation and phosphodiesterase inhibition. *Microvasc. Res.* 50 1–17. 10.1006/mvre.1995.1033 7476570

[B173] Sengillo J. D. Winkler E. A. Walker C. T. Sullivan J. S. Johnson M. Zlokovic B. V. (2013). Deficiency in mural vascular cells coincides with blood-brain barrier disruption in Alzheimer’s disease. *Brain Pathol.* 23 303–310. 10.1111/bpa.12004 23126372 PMC3628957

[B174] Sharma A. Rizky L. Stefanovic N. Tate M. Ritchie R. H. Ward K. W. (2017). The nuclear factor (erythroid-derived 2)-like 2 (Nrf2) activator dh404 protects against diabetes-induced endothelial dysfunction. *Cardiovasc. Diabetol.* 16:33 10.1186/s12933-017-0513-y PMC5335831 28253885

[B175] Shasby D. M. Lind S. E. Shasby S. S. Goldsmith J. C. Hunninghake G. W. (1985). Reversible oxidant-induced increases in albumin transfer across cultured endothelium: alterations in cell shape and calcium homeostasis. *Blood* 65 605–614. 3838256

[B176] Shepro D. Morel N. M. (1993). Pericyte physiology. *FASEB J.* 7 1031–1038. 8370472 10.1096/fasebj.7.11.8370472

[B177] Shih A. Y. Li P. Murphy T. H. (2005). A small-molecule-inducible Nrf2-mediated antioxidant response provides effective prophylaxis against cerebral ischemia in vivo. *J. Neurosci.* 25 10321–10335. 10.1523/JNEUROSCI.4014-05.2005 16267240 PMC6725780

[B178] Shojaee N. Patton W. F. Hechtman H. B. Shepro D. (1999). Myosin translocation in retinal pericytes during free-radical induced apoptosis. *J. Cell Biochem.* 75 118–129. 10462710

[B179] Siflinger-Birnboim A. Goligorsky M. S. Del Vecchio P. J. Malik A. B. (1992). Activation of protein kinase C pathway contributes to hydrogen peroxide-induced increase in endothelial permeability. *Lab. Invest.* 67 24–30. 1378104

[B180] Siflinger-Birnboim A. Lum H. Del Vecchio P. J. Malik A. B. (1996). Involvement of Ca2+ in the H2O2-induced increase in endothelial permeability. *Am. J. Physiol.* 270 L973–L978. 10.1152/ajplung.1996.270.6.L973 8764222

[B181] Sims D. E. (1986). The pericyte–a review. *Tissue Cell* 18 153–174. 3085281 10.1016/0040-8166(86)90026-1

[B182] Sims D. E. Miller F. N. Donald A. Perricone M. A. (1990). Ultrastructure of pericytes in early stages of histamine-induced inflammation. *J. Morphol.* 206 333–342. 10.1002/jmor.1052060310 2280409

[B183] Stadtman E. R. Levine R. L. (2000). Protein oxidation. *Ann. N. Y. Acad. Sci.* 899 191–208. 10863540 10.1111/j.1749-6632.2000.tb06187.x

[B184] Stapor P. C. Sweat R. S. Dashti D. C. Betancourt A. M. Murfee W. L. (2014). Pericyte dynamics during angiogenesis: new insights from new identities. *J. Vasc. Res.* 51 163–174. 10.1159/000362276 24853910 PMC4149862

[B185] Stocker R. Keaney J. F. (2004). Role of oxidative modifications in atherosclerosis. *Physiol. Rev.* 84 1381–1478. 15383655 10.1152/physrev.00047.2003

[B186] Stratman A. N. Davis G. E. (2012). Endothelial cell-pericyte interactions stimulate basement membrane matrix assembly: influence on vascular tube remodeling, maturation, and stabilization. *Microsc. Microanal.* 18 68–80. 10.1017/S1431927611012402 22166617 PMC3919655

[B187] Suematsu M. Schmid-Schonbein G. W. Chavez-Chavez R. H. Yee T. T. Tamatani T. Miyasaka M. (1993). In vivo visualization of oxidative changes in microvessels during neutophil activation. *Am. J. Physiol.* 264 H881–H891. 10.1152/ajpheart.1993.264.3.H881 8096123

[B188] Sugiyama S. Kugiyama K. Aikawa M. Nakamura S. Ogawa H. Libby P. (2004). Hypochlorous acid, a macrophage product, induces endothelial apoptosis and tissue factor expression: involvement of myeloperoxidase-mediated oxidant in plaque erosion and thrombogenesis. *Arterioscler. Thromb. Vasc. Biol.* 24 1309–1314. 10.1161/01.ATV.0000131784.50633.4f 15142860

[B189] Suresh K. Servinsky L. Reyes J. Baksh S. Undem C. Caterina M. (2015). Hydrogen peroxide-induced calcium influx in lung microvascular endothelial cells involves TRPV4. *Am. J. Physiol. Lung Cell. Mol. Physiol.* 309 L1467–L1477. 10.1152/ajplung.00275.2015 26453519 PMC4683318

[B190] Suzuki Y. J. Forman H. J. Sevanian A. (1997). Oxidants as stimulators of signal transduction. *Free Radic. Biol. Med.* 22 269–285. 10.1016/s0891-5849(96)00275-4 8958153

[B191] Szabo C. Cuzzocrea S. Zingarelli B. O’connor M. Salzman A. L. (1997). Endothelial dysfunction in a rat model of endotoxic shock. Importance of the activation of poly (ADP-ribose) synthetase by peroxynitrite. *J. Clin. Invest.* 100 723–735. 10.1172/JCI119585 9239421 PMC508242

[B192] Szabo C. Ischiropoulos H. Radi R. (2007). Peroxynitrite: biochemistry, pathophysiology and development of therapeutics. *Nat. Rev. Drug Discov.* 6 662–680. 10.1038/nrd2222 17667957

[B193] Tailor A. Granger D. N. (2003). Hypercholesterolemia promotes P-selectin-dependent platelet-endothelial cell adhesion in postcapillary venules. *Arterioscler. Thromb. Vasc. Biol.* 23 675–680. 10.1161/01.ATV.0000056742.97580.79 12615684

[B194] Tarantini S. Valcarcel-Ares M. N. Yabluchanskiy A. Tucsek Z. Hertelendy P. Kiss T. (2018). Nrf2 deficiency exacerbates obesity-induced oxidative stress, neurovascular dysfunction, blood-brain barrier disruption, neuroinflammation, amyloidogenic gene expression, and cognitive decline in mice, mimicking the aging phenotype. *J. Gerontol. A Biol. Sci. Med. Sci.* 73 853–863. 10.1093/gerona/glx177 29905772 PMC6001893

[B195] Tatsumi T. Fliss H. (1994). Hypochlorous acid and chloramines increase endothelial permeability: possible involvement of cellular zinc. *Am. J. Physiol.* 267 H1597–H1607. 10.1152/ajpheart.1994.267.4.H1597 7943407

[B196] Thannickal V. J. Fanburg B. L. (2000). Reactive oxygen species in cell signaling. *Am. J. Physiol. Lung Cell. Mol. Physiol.* 279 L1005–L1028. 11076791 10.1152/ajplung.2000.279.6.L1005

[B197] Tian R. Ding Y. Peng Y. Y. Lu N. (2017). Myeloperoxidase amplified high glucose-induced endothelial dysfunction in vasculature: role of NADPH oxidase and hypochlorous acid. *Biochem. Biophys. Res. Commun.* 484 572–578. 10.1016/j.bbrc.2017.01.132 28131839

[B198] Tinsley J. H. Ustinova E. E. Xu W. S. Y. Yuan S. Y. (2002). Src-dependent, neutrophil-mediated vascular hyperpermeability and β-catenin modification. *Am. J. Physiol. Cell. Physiol.* 283, C1745–C1751. 10.1152/ajpcell.00230.2002 12388068

[B199] Tiyerili V. Camara B. Becher M. U. Schrickel J. W. Lutjohann D. Mollenhauer M. (2016). Neutrophil-derived myeloperoxidase promotes atherogenesis and neointima formation in mice. *Int. J. Cardiol.* 204 29–36. 10.1016/j.ijcard.2015.11.128 26655530

[B200] Ungvari Z. Bailey-Downs L. Gautam T. Jimenez R. Losonczy G. Zhang C. (2011a). Adaptive induction of NF-E2-related factor-2-driven antioxidant genes in endothelial cells in response to hyperglycemia. *Am. J. Physiol. Heart Circ. Physiol.* 300 H1133–H1140. 10.1152/ajpheart.00402.2010 21217061 PMC3075025

[B201] Ungvari Z. Bailey-Downs L. Sosnowska D. Gautam T. Koncz P. Losonczy G. (2011b). Vascular oxidative stress in aging: a homeostatic failure due to dysregulation of NRF2-mediated antioxidant response. *Am. J. Physiol. Heart Circ. Physiol.* 301 H363–H372. 10.1152/ajpheart.01134.2010 21602469 PMC3154665

[B202] Usatyuk P. V. Natarajan V. (2004). Role of mitogen-activated protein kinases in 4-hydroxy-2-nonenal-induced actin remodeling and barrier function in endothelial cells. *J. Biol. Chem.* 279 11789–11797. 10.1074/jbc.M311184200 14699126

[B203] Valko M. Leibfritz D. Moncol J. Cronin M. T. Mazur M. Telser J. (2007). Free radicals and antioxidants in normal physiological functions and human disease. *Int. J. Biochem. Cell Biol.* 39 44–84. 10.1016/j.biocel.2006.07.001 16978905

[B204] van Dalen C. J. Whitehouse M. W. Winterbourn C. C. Kettle A. J. (1997). Thiocyanate and chloride as competing substrates for myeloperoxidase. *Biochem. J.* 327(Pt 2), 487–492. 10.1042/bj3270487 9359420 PMC1218820

[B205] van der Veen B. S. De Winther M. P. Heeringa P. (2009). Myeloperoxidase: molecular mechanisms of action and their relevance to human health and disease. *Antioxid. Redox Signal.* 11 2899–2937. 10.1089/ars.2009.2538 19622015

[B206] van Dijk C. G. Nieuweboer F. E. Pei J. Y. Xu Y. J. Burgisser P. Van Mulligen E. (2015). The complex mural cell: pericyte function in health and disease. *Int. J. Cardiol.* 190 75–89. 10.1016/j.ijcard.2015.03.258 25918055

[B207] van Leeuwen H. J. Van Der Tol M. Van Strijp J. A. Verhoef J. Van Kessel K. P. (2005). The role of tumour necrosis factor in the kinetics of lipopolysaccharide-mediated neutrophil priming in whole blood. *Clin. Exp. Immunol.* 140 65–72. 10.1111/j.1365-2249.2005.02748.x 15762876 PMC1809339

[B208] van Leeuwen M. Gijbels M. J. Duijvestijn A. Smook M. Van De Gaar M. J. Heeringa P. (2008). Accumulation of myeloperoxidase-positive neutrophils in atherosclerotic lesions in LDLR-/- mice. *Arterioscler. Thromb. Vasc. Biol.* 28 84–89. 10.1161/ATVBAHA.107.154807 17991873

[B209] Vepa S. Scribner W. M. Natarajan V. (1997). Activation of protein phosphorylation by oxidants in vascular endothelial cells: identification of tyrosine phosphorylation of caveolin. *Free Radic. Biol. Med.* 22 25–35. 10.1016/s0891-5849(96)00241-9 8958127

[B210] Volk T. Hensel M. Kox W. J. (1997). Transient Ca2+ changes in endothelial cells induced by low doses of reactive oxygen species: role of hydrogen peroxide. *Mol. Cell Biochem.* 171 11–21. 10.1023/a:1006886215193 9201690

[B211] von Tell D. Armulik A. Betsholtz C. (2006). Pericytes and vascular stability. *Exp. Cell Res.* 312 623–629. 16303125 10.1016/j.yexcr.2005.10.019

[B212] Warmke N. Griffin K. J. Cubbon R. M. (2016). Pericytes in diabetes-associated vascular disease. *J. Diabetes Complicat.* 30 1643–1650. 27592245 10.1016/j.jdiacomp.2016.08.005

[B213] Wenzel P. Mollnau H. Oelze M. Schulz E. Wickramanayake J. M. Muller J. (2008). First evidence for a crosstalk between mitochondrial and NADPH oxidase-derived reactive oxygen species in nitroglycerin-triggered vascular dysfunction. *Antioxid. Redox Signal* 10 1435–1447. 10.1089/ars.2007.1969 18522491

[B214] Wink D. A. Cook J. A. Pacelli R. Liebmann J. Krishna M. C. Mitchell J. B. (1995). Nitric oxide (NO) protects against cellular damage by reactive oxygen species. *Toxicol. Lett.* 82-83 221–226. 10.1016/0378-4274(95)03557-5 8597056

[B215] Wong N. D. Gransar H. Narula J. Shaw L. Moon J. H. Miranda-Peats R. (2009). Myeloperoxidase, subclinical atherosclerosis, and cardiovascular disease events. *JACC Cardiovasc. Imaging* 2 1093–1099. 10.1016/j.jcmg.2009.05.012 19761988

[B216] Wu F. Wilson J. X. (2009). Peroxynitrite-dependent activation of protein phosphatase type 2A mediates microvascular endothelial barrier dysfunction. *Cardiovasc. Res.* 81 38–45. 10.1093/cvr/cvn246 18791203 PMC2605194

[B217] Wu H. M. Huang Q. Yuan Y. Granger H. J. (1996). VEGF induces NO-dependent hyperpermeability in coronary venules. *Am. J. Physiol.* 271 H2735–H2739. 8997338 10.1152/ajpheart.1996.271.6.H2735

[B218] Xia X. Lapenna K. B. Zhang Y. He P. (2018). Nrf2 deficiency exacerbates oxidative stress and microvessel susceptibility to inflammation in diabetic rats. *FASEB J.* 32 708–706.

[B219] Xia Y. Dawson V. L. Dawson T. M. Snyder S. H. Zweier J. L. (1996). Nitric oxide synthase generates superoxide and nitric oxide in arginine-depleted cells leading to peroxynitrite-mediated cellular injury. *Proc. Natl. Acad. Sci. U.S.A.* 93 6770–6774. 10.1073/pnas.93.13.6770 8692893 PMC39102

[B220] Xu S. Zhou X. Yuan D. Xu Y. He P. (2013). Caveolin-1 scaffolding domain promotes leukocyte adhesion by reduced basal endothelial nitric oxide-mediated ICAM-1 phosphorylation in rat mesenteric venules. *Am. J. Physiol. Heart Circ. Physiol.* 305 H1484–H1493. 10.1152/ajpheart.00382.2013 24043249 PMC3840264

[B221] Yang J. Cheng Y. Ji R. Zhang C. (2006). Novel model of inflammatory neointima formation reveals a potential role of myeloperoxidase in neointimal hyperplasia. *Am. J. Physiol. Heart Circ. Physiol.* 291 H3087–H3093. 10.1152/ajpheart.00412.2006 16844918

[B222] Yang Z. Zhang A. Altura B. T. Altura B. M. (1999). Hydrogen peroxide-induced endothelium-dependent relaxation of rat aorta involvement of Ca2+ and other cellular metabolites. *Gen. Pharmacol.* 33 325–336. 10.1016/s0306-3623(99)00019-1 10523071

[B223] Yla-Herttuala S. (1999). Oxidized LDL and atherogenesis. *Ann. N. Y. Acad. Sci.* 874 134–137. 10415527 10.1111/j.1749-6632.1999.tb09231.x

[B224] Yu H. Wang M. Wang D. Kalogeris T. J. Mchowat J. Ford D. A. (2019). Chlorinated Lipids Elicit Inflammatory Responses in vitro and in vivo. *Shock* 51 114–122. 10.1097/SHK.0000000000001112 29394241 PMC6070441

[B225] Yuan D. He P. (2012). Vascular remodeling alters adhesion protein and cytoskeleton reactions to inflammatory stimuli resulting in enhanced permeability increases in rat venules. *J. Appl. Physiol.* 113 1110–1120. 10.1152/japplphysiol.00102.2012 22837164 PMC3487498

[B226] Yuan Y. Granger H. J. Zawieja D. C. Defily D. V. Chilian W. M. (1993). Histamine increases venular permeability via a phospholipase C-NO synthase-guanylate cyclase cascade. *Am. J. Physiol.* 264 H1734–H1739. 10.1152/ajpheart.1993.264.5.H1734 7684577

[B227] Zeng M. Zhang H. Lowell C. He P. (2002). Tumor necrosis factor-alpha-induced leukocyte adhesion and microvessel permeability. *Am. J. Physiol. Heart Circ. Physiol.* 283 H2420–H2430. 10.1152/ajpheart.00787.2001 12388263

[B228] Zhang C. Patel R. Eiserich J. P. Zhou F. Kelpke S. Ma W. (2001). Endothelial dysfunction is induced by proinflammatory oxidant hypochlorous acid. *Am. J. Physiol. Heart Circ. Physiol.* 281 H1469–H1475. 10.1152/ajpheart.2001.281.4.H1469 11557534

[B229] Zhang R. Brennan M. L. Fu X. Aviles R. J. Pearce G. L. Penn M. S. (2001). Association between myeloperoxidase levels and risk of coronary artery disease. *JAMA* 286 2136–2142. 10.1001/jama.286.17.2136 11694155

[B230] Zhao J. Moore A. N. Redell J. B. Dash P. K. (2007). Enhancing expression of Nrf2-driven genes protects the blood brain barrier after brain injury. *J. Neurosci.* 27 10240–10248. 10.1523/JNEUROSCI.1683-07.2007 17881530 PMC6672672

[B231] Zhou X. He P. (2010). Endothelial [Ca2+]i and caveolin-1 antagonistically regulate eNOS activity and microvessel permeability in rat venules. *Cardiovasc. Res.* 87 340–347. 10.1093/cvr/cvq006 20080986 PMC2895537

[B232] Zhou X. Qian Y. Yuan D. Feng Q. He P. (2019). H_2_O_2_-induced microvessel barrier dysfunction: the interplay between reactive oxygen species, nitric oxide, and peroxynitrite. *Physiol. Rep.* 7:e14206. 10.14814/phy2.14206 PMC6709418 31448579

[B233] Zhou X. Wen K. Yuan D. Ai L. He P. (2009). Calcium influx-dependent differential actions of superoxide and hydrogen peroxide on microvessel permeability. *Am. J. Physiol. Heart Circ. Physiol.* 296 H1096–H1107. 10.1152/ajpheart.01037.2008 19201997 PMC2670695

[B234] Zhou X. Yuan D. Wang M. He P. (2013). H2O2-induced endothelial NO production contributes to vascular cell apoptosis and increased permeability in rat venules. *Am. J. Physiol. Heart Circ. Physiol.* 304 H82–H93. 10.1152/ajpheart.00300.2012 23086988 PMC3543683

[B235] Zhou X. P. He P. N. (2011). Temporal and spatial correlation of platelet-activating factor-induced increases in endothelial [Ca2+](i), nitric oxide, and gap formation in intact venules. *Am. J. Physiol. Heart Circ. Physiol.* 301 H1788–H1797. 10.1152/ajpheart.00599.2011 21873500 PMC3213973

[B236] Zhu L. Castranova V. He P. (2005). fMLP-stimulated neutrophils increase endothelial [Ca2+]i and microvessel permeability in the absence of adhesion: role of reactive oxygen species. *Am. J. Physiol. Heart Circ. Physiol.* 288 H1331–H1338. 10.1152/ajpheart.00802.2004 15498822

[B237] Zhu L. He P. (2005). Platelet-activating factor increases endothelial [Ca2+]i and NO production in individually perfused intact microvessels. *Am. J. Physiol. Heart Circ. Physiol.* 288 H2869–H2877. 10.1152/ajpheart.01080.2004 15665052

[B238] Zhu L. He P. (2006). fMLP-stimulated release of reactive oxygen species from adherent leukocytes increases microvessel permeability. *Am. J. Physiol. Heart Circ. Physiol.* 290 H365–H372. 10.1152/ajpheart.00812.2005 16155097

[B239] Zinkevich N. S. Gutterman D. D. (2011). ROS-induced ROS release in vascular biology: redox-redox signaling. *Am. J. Physiol. Heart Circ. Physiol.* 301 H647–H653. 10.1152/ajpheart.01271.2010 21685266 PMC3191081

[B240] Zou M. H. Hou X. Y. Shi C. M. Nagata D. Walsh K. Cohen R. A. (2002). Modulation by peroxynitrite of Akt- and AMP-activated kinase-dependent Ser1179 phosphorylation of endothelial nitric oxide synthase. *J. Biol. Chem.* 277 32552–32557. 10.1074/jbc.M204512200 12107173

[B241] Zweier J. L. Talukder M. A. (2006). The role of oxidants and free radicals in reperfusion injury. *Cardiovasc. Res.* 70 181–190. 10.1016/j.cardiores.2006.02.025 16580655

